# All‐Natural Immunomodulatory Bioadhesive Hydrogel Promotes Angiogenesis and Diabetic Wound Healing by Regulating Macrophage Heterogeneity

**DOI:** 10.1002/advs.202206771

**Published:** 2023-03-02

**Authors:** Ya‐Jun Fu, Yi‐Feng Shi, Li‐Ya Wang, Yi‐Fan Zhao, Rao‐Kaijuan Wang, Kai Li, Shu‐Ting Zhang, Xiang‐Jun Zha, Wei Wang, Xing Zhao, Wei Yang

**Affiliations:** ^1^ College of Polymer Science and Engineering Sichuan University Chengdu 610065 P. R. China; ^2^ Department of Neurosurgery West China Hospital Sichuan University Chengdu 610041 P. R. China; ^3^ Department of Nephrology West China Hospital Sichuan University Chengdu 610041 P. R. China; ^4^ Department of Orthodontics West China Hospital of Stomatology Sichuan University Chengdu 610032 P. R. China; ^5^ Department of Thoracic Oncology West China Hospital Sichuan University Chengdu 610041 P. R. China; ^6^ Laboratory of Liver Transplantation West China Hospital Sichuan University Chengdu 610041 P. R. China

**Keywords:** all‐natural hydrogel, angiogenesis, diabetic wound healing, immunomodulation

## Abstract

Macrophages are highly heterogeneous and exhibit a diversity of functions and phenotypes. They can be divided into pro‐inflammatory macrophages (M1) and anti‐inflammatory macrophages (M2). Diabetic wounds are characterized by a prolonged inflammatory phase and difficulty in healing due to the accumulation of pro‐inflammatory (M1) macrophages in the wound. Therefore, hydrogel dressings with macrophage heterogeneity regulation function hold great promise in promoting diabetic wound healing in clinical applications. However, the precise conversion of pro‐inflammatory M1 to anti‐inflammatory M2 macrophages by simple and biosafe approaches is still a great challenge. Here, an all‐natural hydrogel with the ability to regulate macrophage heterogeneity is developed to promote angiogenesis and diabetic wound healing. The protocatechuic aldehyde hybridized collagen‐based all‐natural hydrogel exhibits good bioadhesive and antibacterial properties as well as reactive oxygen species scavenging ability. More importantly, the hydrogel is able to convert M1 macrophages into M2 macrophages without the need for any additional ingredients or external intervention. This simple and safe immunomodulatory approach shows great application potential for shortening the inflammatory phase of diabetic wound repair and accelerating wound healing.

## Graphical abstract

An all‐natural hydrogel composed of small molecules pro‐catechol and collagen is developed to promote diabetic wound healing by modulating macrophage heterogeneity. The hydrogel exhibits good bioadhesive, antibacterial, and reactive oxygen species scavenging abilities. In vitro and in vivo experiments show that the hydrogel is able to promote the conversion of pro‐inflammatory (M1) macrophages to anti‐inflammatory (M2) macrophages and the expression of anti‐inflammatory factors.

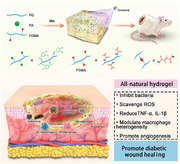

## 1 Introduction

With the increase of the aging and obese population, the prevalence of diabetes is rising every year and the treatment of diabetic wounds has become a major challenge for the global health care system.^[^ ^1^ ^]^ The wound healing process is mainly divided into four phases: hemostasis, inflammation, proliferation, and remodeling.^[^ ^2^ ^]^ In diabetic wounds, a series of complications arise due to the specific high‐glucose environment that disrupts the physiological cascade response of wound healing and makes the polarization of macrophage from pro‐inflammatory (M1) to anti‐inflammatory (M2) phenotype very difficult.^[^ ^3^ ^]^ A large number of M1 macrophages accumulate in the wound, releasing pro‐inflammatory cytokines such as tumor necrosis factor (TNF‐*α*) and generating reactive oxygen species (ROS).^[^ ^4^ ^]^ As a result, an oxidative stress and cellular damage microenvironment is generated in the wound, leading to a prolonged inflammatory phase and vasculopathy.^[^ ^5^ ^]^ Ultimately, it will lead to non‐healing wounds and increase the risk of infection, causing a vicious cycle. Therefore, the regulation of the immune microenvironment and promotion of angiogenesis are essential for diabetic wound healing. Immunomodulation induces the expression of transforming and proliferative cytokines that will have a property to promote angiogenesis. However, the induction of angiogenesis by in situ immunomodulation is still difficult to achieve.^[^ ^6^ ^]^ Thus, regulating the wound microenvironment by modulating macrophages heterogeneity for immunomodulation is a prospective strategy for the treatment of diabetic chronic wounds.

Hydrogels have become the most widely studied wound dressing in recent years because of their extracellular matrix (ECM) structure, good biocompatibility and the ability to keep the wound moist for a long time.^[^ ^7^ ^]^ The strategies for regulating macrophage polarization with hydrogels can be divided into two categories. One category uses hydrogels to deliver cells, proteins, growth factors, genes, and immunomodulatory active substances to regulate macrophage polarization.^[^ ^7^, ^8^ ^]^ The other category relies on the properties of hydrogels themselves. For example, studies have shown that low molecular weight hyaluronic acid (HA) hydrogels stimulate inflammation responses, while collagen‐based hydrogels can promote an anti‐inflammatory phenotype by binding integrin *α*2*β*1.^[^ ^4^, ^9^ ^]^ These strategies are costly and require complex production processes, and the immunomodulatory capacity of a single hydrogel matrix is not sufficiently effective. Strategically, hydrogel matrices should have the capability to promote anti‐inflammatory conversion of macrophages and need to contain intrinsic immunomodulatory properties. Consequently, a hydrogel matrix needs to be featured with good bioactivity,^[^ ^10^ ^]^ and alternatively, naturally sourced hydrogels generally have better bioactivity than synthetic hydrogels.^[^ ^11^ ^]^ Moreover, they have the advantages of sufficient resources and excellent biocompatibility.^[^ ^10a^ ^]^ In order to achieve an intrinsic immunomodulatory capacity, a simple superimposition of bioactive factors is not sufficient and may cause complications. In recent years, many reported natural small molecules, including green tea derivatives, glycyrrhetinic acid, curcumin, ginsenosides and so on, can play a multifunctional therapeutic role.^[^ ^12^ ^]^ These natural small molecules tend to form some physical or chemical interactions with the hydrogel matrix, and hybridized hydrogels not only have the ability to promote healing of complex wound, but also feature low cost and good biosafety. Therefore, these natural small molecules may provide a new alternative for the intrinsic immunomodulation of hydrogels.

Protocatechuic aldehyde (PA) is a natural compound derived from *Salvia miltiorrhiza* with an unique phenolic aldehyde structure compared to natural polyphenols such as tannic acid and gallic acid with only phenol structure.^[^ ^13^ ^]^ Therefore, it can form reversible Schiff base bonds with amino groups.^[^ ^2b^ ^]^ Studies have shown that PA has good anti‐inflammatory and antibacterial effects and has been used in clinical applications as an active ingredient in the coronary heart disease drug.^[^ ^14^ ^]^ PA has also been used in the treatment of chronic nephritis, vascular disease, etc., displaying good biocompatibility.^[^ ^13b^ ^]^ In addition, PA contains catechol groups with the ability to scavenge ROS.^[^ ^2b^ ^]^ These good properties make PA highly suitable for diabetic wound treatment. However, the mechanism by which PA modulates immune microenvironment at diabetic wounds to accelerate wound healing remains to be investigated. Meanwhile, fish gelatin (FG) was a good candidate of the hydrogel matrix, which contains arginine‐glycine‐aspartate (RGD) peptide sequence that can interact with integrin receptors so that it has the ability to promote anti‐inflammatory transformation.^[^ ^8^, ^9^ ^]^

Here, an all‐natural hydrogel dressing was designed to modulate the immune microenvironment of diabetic wound by regulating macrophages heterogeneity and validate the importance of immunomodulation for vascular regeneration in the wound. The all‐natural hydrogel (FGMA/FG/PA) consists of a physical entanglement network formed by the FG/PA, and a chemical crosslinking network by photo crosslinked methacrylate gelatin (FGMA). The physically entangled FG/PA network can adapt to the deformation during wound movement, and the photo‐crosslinked FGMA can increase the hydrogel strength to provide a better protection for the wound. With the cooperative effect of FG and PA, the hydrogel shows strong bioadhesive property to physically close the wounds and reduce external infection, while the introduction of PA endows the hydrogel with intrinsic chemical antibacterial effect and ROS scavenging ability, thus reducing inflammation of the wound. Because of these, the all‐natural hydrogel is capable to promote the polarization of macrophages toward the M2 phenotype without adding any active ingredient. Since M2 macrophages release transforming growth factor (TGF‐*β*) and vascular endothelial growth factor (VEGF), the hydrogel is able to promote angiogenesis through immunomodulation. So, the all‐natural hydrogel dressing shows great potential for application in clinical treatment of diabetic wounds due to its bioactivity, biosafety, simple preparation, and abundance of raw materials.

## 2 Results and Discussion

### 2.1 Preparation of the FGMA/FG/PA Hydrogel

**Figure 1:**
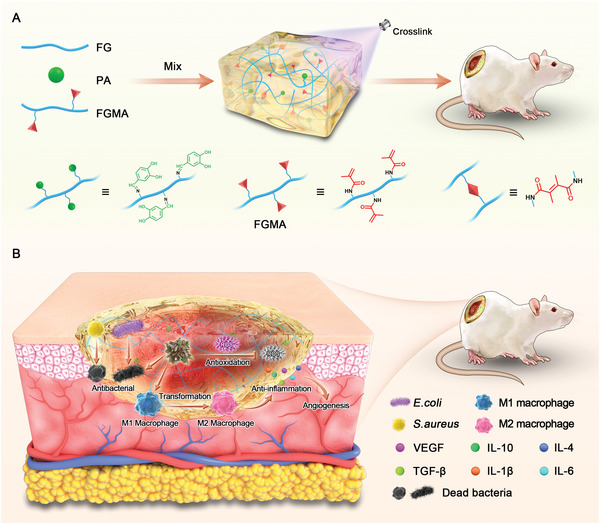
A multifunctional all‐natural hydrogel as an immunomodulatory dressing for diabetic wound healing. A) Schematic illustration of the preparation of the all‐natural hydrogel and B) the immunoregulation in diabetic wound healing.

The synthetic procedures of the all‐natural hydrogel (FGMA/FG/PA) and further applications in diabetic chronic wound dressing are depicted in **Figure** **1**. Briefly, the three main components, FGMA, FG, and PA, are mixed to form a preliminary physically entangled network by combining PA with FG in a Schiff base reaction. Next, FGMA polymerizes via free radical polymerization under visible light to further form a chemical cross‐linked network.

In this work, a photo‐crosslinkable FG prepolymer (FGMA) was designed and synthesized by modifying FG with reactive methacrylate groups. The successful conjugation was initially confirmed by the blue shift of the amide II band in the Fourier transform infrared spectrum (FTIR spectrum) as shown in Figure S1A, Supporting Information.^[^ ^15^ ^]^ Similarly, the proton peak of methyl (—CH=CH_2_) appeared at 5.3 and 5.6 ppm as seen in proton nuclear magnetic resonance (^1^H‐NMR) (Figure S1B, Supporting Information), indicating the successful introduction of double bonds into FG and the successful synthesis of FGMA.^[^ ^16^ ^]^ From the ratio of the peak reduction at 2.9 ppm (due to the chemical bonding between the lysine residue and the acrylate group), the methacrylating degree of FGMA is calculated to be 50.2%.^[^ ^17^ ^]^ Similarly, in the ^1^H‐NMR spectrum of FG/PA, new peaks at 8.4 ppm (—CH=N) and at 7.3–7.4 (C—H) and 6.9 ppm (O—H) belonging to PA appeared compared to FG, confirming the Schiff base reaction of PA and FG (Figure S1C, Supporting Information).^[^ ^18^ ^]^ Finally, the mixture solution of FG, PA, and FGMA was cured under 405 nm visible light to form a composite hydrogel wound dressing (Figure S1D, Supporting Information).

### 2.2 Characterization of the FGMA/FG/PA Hydrogel

For wound dressings, porous structures that promote cell migration and adhesion are essential,^[^ ^19^ ^]^ so the morphological structure of the hydrogels was examined. The microstructures of the FGMA and FGMA/FG/PA hydrogels were observed by scanning electron microscopy (SEM), and both hydrogels have interconnected 3D porous structures (Figure S2A, Supporting Information). Compared with FGMA hydrogels, the introduction of FG/PA results in thicker pore walls, more uniform pores, and reduced pore size. This is because the introduction of FG/PA increases the physical entanglement network and increases the interaction of molecular chains in the hydrogel matrix. Thus makes the hydrogels more conducive to providing support for cell adhesion.

**Figure 2:**
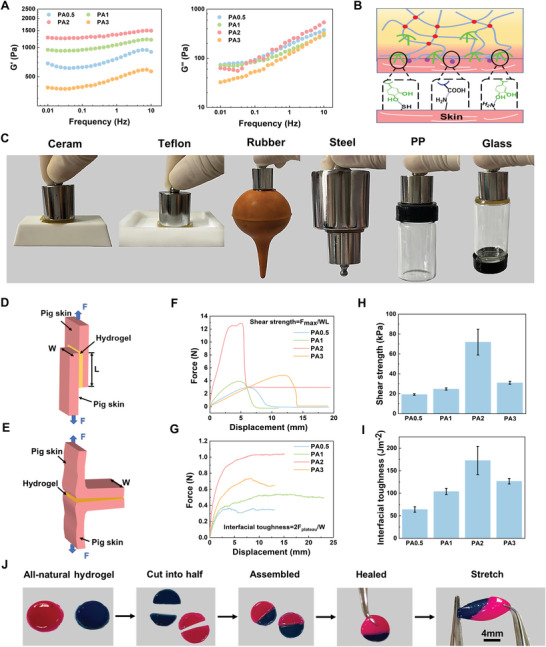
Rheological, adhesion, and self‐healing properties of the all‐natural hydrogels. A) Rheological properties of the hydrogels at constant strain at a frequency of 0.01–10 Hz. B) Diagram of the adhesion mechanism between hydrogel and skin. C) Digital images demonstrating the macroscopic adhesion of the hydrogel to various substrates. Schematic diagram of D) shear strength based on the standard lap‐shear test and E) interfacial toughness based on 180‐degree test. F) Shear strength profiles and G) interfacial toughness profiles of the porcine skin bonded by the FGMA/FG/PA hydrogel. H) The shear strength and I) interfacial toughness of the hydrogels on porcine skin. J) Photograph of the self‐healing process for the FGMA/FG/PA hydrogel.

Due to the long‐term open wounds in diabetes, the ideal wound dressing needs to be strong enough to avoid secondary injuries and also be able to accommodate the deformation of the wound during movement. Therefore, the mechanical properties of the hydrogel were tested. The storage modulus (*G*′) is consistently greater than the loss modulus (*G*″) when the strain range is 0.01–1% (Figure S2B,C, Supporting Information), confirming the elastic behavior of the hydrogel.^[^ ^12b^ ^]^ Since the effect of PA content on the mechanical properties of hydrogels is crucial, frequency scanning experiments were conducted (**Figure** **2**A). At a frequency of 0.01–10 Hz, the FGMA/FG/PA hydrogel with 2% PA shows the highest energy storage modulus (*G*′) and also a higher loss modulus (*G*″). This indicates that FGMA/FG/PA hydrogel with 2% PA has a unique viscoelastic behavior, and the high energy storage modulus shows good elasticity and mechanical stability, while the higher loss modulus means that the materials can dissipate energy to avoid secondary damage during wound movement.^[^ ^20^ ^]^ The FGMA/FG/PA hydrogel can adapt to the bending of the finger at different angles and can fit well on the finger without breaking (Figure S2D, Supporting Information), which also indicates that the FGMA/FG/PA hydrogel has good mechanical strength and the ability to adapt the wound deformation.

The ideal diabetic wound dressings should have high tissue adhesion strength for good wound closure and reducing the invasion of external bacteria. Studies have shown that materials rich in hydrogen bonds and catechol groups exhibit better adhesion to tissues as well as various substrates.^[^ ^12^, ^21^ ^]^ We also demonstrated that the FGMA/FG/PA hydrogel can adhere to a variety of substrates^[^ ^22^ ^]^ such as fingers, ceramics, polytetrafluoroethylene, rubber, iron, plastic, and glass (Figure 2C; Figure S2D, Supporting Information). The reason for this is that FG is rich in amino and carboxyl groups as well as hydrophobic fragments, and the introduction of catechol groups further increases the non‐covalent interactions of the hydrogel with various materials, which makes the FGMA/FG/PA hydrogel have good bioadhesion.^[^ ^23^ ^]^ To simulate the adhesion of the FGMA/FG/PA hydrogel to tissues, macroscopic adhesion performance tests were performed by using pigskin. Its adhesion performance was evaluated by lap shear and peel experiments,^[^ ^24^ ^]^ and the schematic diagrams are shown in Figures 2D,E. The shear strength and interfacial toughness of the FGMA/FG/PA hydrogel with different PA contents were calculated from the obtained force–displacement curves (Figure 2F,G). As shown in Figures 2H,I, the shear strength and interfacial toughness are as high as 71.93 kPa and 172.66 J m^−2^ respectively, when the PA content is 2%. This is because the test results of adhesion properties are also influenced by the mechanical properties of the hydrogel materials. If the hydrogel cannot dissipate the energy generated by shear and peel and appears to be damaged on the matrix, it will lead to a decrease in the bonding force. Through the rheological performance test (Figure 2A), it can be found that the PA2 hydrogel has a larger energy storage modulus, so it is more resistant to external deformation and less susceptible to damage. Therefore, the PA2 hydrogel has better adhesion properties. The adhesion properties are better compared to commercial adhesive dressings (shear strength is about 20 kPa and interfacial toughness is about 100 J m^−2^).^[^ ^24a^ ^]^ The mechanism of strong adhesion to tissue is that the strong non‐covalent interaction of the catechol groups with the thiol and amine groups and the hydrogen bonding of the large number of carboxyl groups in FG with the amine groups on the tissue (Figure 2B). To further verify the practical application capability of the FGMA/FG/PA hydrogel, the hydrogel was cut into two halves to simulate fracture during use and to study the self‐healing properties after fracture.^[^ ^25^ ^]^ The healing was completed after the two parts of the broken hydrogel contacted for 2 minutes, and then the healed hydrogel was stretched and it was able to withstand a tensile force, which indicated its good practicality (Figure 2J).

The stability and biodegradability of the hydrogel dressings are crucial in practical applications. The FGMA/FG/PA hydrogel has a lower swelling ratio than FGMA hydrogel (Figure S2E, Supporting Information), and its low swelling ratio prevents detachment from the wound after implantation. In vitro degradation was investigated by immersing FGMA and FGMA/FG/PA hydrogels in type II collagenase solution (2 µg mL^−1^). FGMA/FG/PA hydrogel showed a better stability than FGMA hydrogel, which lost 50% of its weight on day 10 and 95% of its weight on day 15 (Figure S2F, Supporting Information). The FGMA/FG/PA hydrogel can be completely degraded under physiological conditions. And the FGMA/FG/PA hydrogel with low swelling ratio and slow biodegradation rate is suitable for wound dressing applications. This is due to the smaller pore size and thicker pore walls of FGMA/FG/PA hydrogel, which prevent greater swelling of the hydrogel and collagenase diffusion. It remains stable in the early stages of wound healing to provide a moist environment for the wound and gradually degrades at a later stage to allow regeneration of new tissue. The removability of dressings is also crucial in the clinical use. As the PA in the hydrogel is released and the hydrogel begins to degrade, its adhesion ability will gradually diminish. At this point, it can be easily removed by using physiological saline wet compresses.

### 2.3 In Vitro Antibacterial Properties and Biocompatibility Evaluation

**Figure 3:**
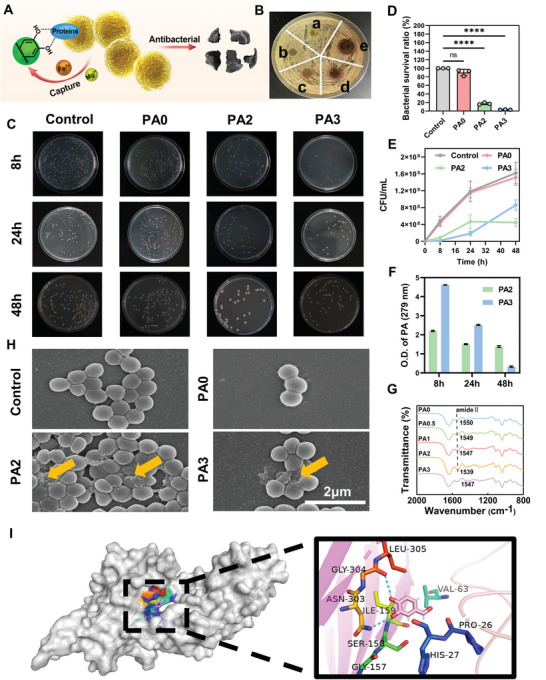
Antibacterial performance of the all‐natural FGMA/FG/PA hydrogel against *S. aureus*. A) Diagram on the antibacterial mechanism of PA. B) Zone of inhibition of the FGMA/FG/PA hydrogels (a: PA0; b: PA0.5; c: PA1; d: PA2; e: PA3). C) Image of bacteria clones on culture plates against *S. aureus* after incubation for 8 h, 24 h, and 48 h at 37 °C. D) Bacterial viability against *S. aureus* after incubation for 8 h at 37 °C (****, *p* < 0.0001, *n* = 3). E) Proliferation curve of *S. aureus* after treatment with FGMA/FG/PA hydrogels. F) Absorbance of PA released from FGMA/FG/PA in PBS. G) FTIR spectra of the FGMA/FG/PA hydrogels. H) SEM images of bacterial morphology after incubation for 8 h at 37 °C (scale bar: 3 µm). I) Simulation of the interaction of PA with bacterial membrane proteins.

In diabetic wounds, the complex wound microenvironment is susceptible to bacterial invasion.^[^ ^25^, ^26^ ^]^ Therefore, hydrogel wound dressings not only need to have good mechanical strength and tissue adhesion properties to reduce secondary infection, but also need to have antibacterial properties to defend against the invasion of external bacteria. Therefore, the antibacterial properties of the all‐natural FGMA/FG/PA hydrogel were investigated with *Staphylococcus aureus* and *Escherichia coli*, as shown in **Figure** **3** and Figure S4, Supporting Information. Many studies have reported the use of polyphenols in antibacterial applications, and the main acting groups are the catechol groups.^[^ ^27^ ^]^ The antibacterial mechanism of the catechol functional group is that the catechol group interacts with bacterial surface membrane proteins and traps nutrients which are required for bacterial proliferation, such as Mg^2+^ and Fe^3+^, thereby inhibiting the activity and proliferation of bacteria, as shown in Figure 3A.^[^ ^27^ ^]^ For the FGMA/FG/PA hydrogel, the main factor affecting the antibacterial effect is the PA content. The inhibition circle experiments verified the weak bacterial inhibition efficiency of the FGMA/FG/PA hydrogel at 0%, 0.5%, and 1% PA content (Figure 3B). Considering the mechanical properties and tissue adhesion ability, the FGMA/FG/PA hydrogel with PA contents of 2% and 3% were selected for the subsequent antibacterial performance study.

The antibacterial properties of the hydrogel were further quantified by plate counting method. Subsequently, after 8 h co‐culture, the FGMA/FG/PA hydrogel containing PA shows good antibacterial effect, with the survival rate of *S. aureus* being below 20%, and the survival rate of *E. coli* in the FGMA/FG/PA hydrogel group significantly reduced compared to the control group (Figure 3C,D; Figure S4A,C, Supporting Information). Surprisingly, the antibacterial effect of FGMA/FG/PA hydrogel with 2% PA at 48 h is better than that of 3% PA (Figure 3E). This can be explained by the slower release of PA in FGMA/FG/PA hydrogel with 2% PA. PA contains a benzene ring, which results in a strong absorption peak at 279 nm. We measured the absorbance of PA by UV absorption spectrophotometry in response to its release. As shown in Figure 3F, PA3 releases much less PA than PA2 at 48 h. It indicates that the FGMA/FG/PA hydrogel with 2% PA has PA retarding ability along with more sustained bacterial inhibition effect. The release rate of PA depends on the strength of the interaction between PA and hydrogel matrix, so FTIR spectroscopy test was performed to verify the variation of the interactions. As shown in Figure 3G, with the increase of PA content, the amide II band shows a red shift, which is attributed to the hydroxyl groups on the benzene ring of PA forming hydrogen bonds with the N—H groups of FG.^[^ ^23^ ^]^ But, at 3% PA content, the amide II band is blue‐shifted. This means that the strength of hydrogen bonding interaction increases with increasing PA content from 0% to 2%, while at 3% PA content, the hydrogen bonding interaction weakens. The morphology of bacteria after treating with the hydrogels was examined by SEM. The hydrogels containing PA show some degree of damage to the morphology of both bacteria, which appeared either depressed or crumpled (Figure 3H; Figure S4B, Supporting Information). This is due to the interaction between PA and the outer membrane of bacteria, resulting in the change of bacterial morphology.

Hydrogel wound dressings can provide a good microenvironment for wound repair if they have the ability to resist infection, which requires that the hydrogel can reduce the adhesion and binding of bacteria to normal cells.^[^ ^28^ ^]^ In this study, the FGMA/FG/PA hydrogel shows good anti‐bacterial adhesion ability (Figures S3 and S4D, Supporting Information), so it can reduce the adhesion of bacteria and decrease the risk of infection. In addition, the binding interaction between the FGMA/FG/PA hydrogel and *S. aureus* fibronectin‐binding proteins (FnBPs) was analyzed by molecular docking.^[^ ^29^ ^]^ Consequently, PA forms three hydrogen bonds with FnBPs, as shown in Figure 3I, and the three hydrogen bonds are GLY‐304 (2.5 Å), ASN‐303 (3.2 Å), and ILE‐159 (3.2 Å). Since FnBPs are targets for bacterial binding to host cells, they affect bacterial adhesion and invasion of host cells.^[^ ^30^ ^]^ Therefore, the FGMA/FG/PA hydrogel manifests anti‐infective ability through hydrogen bonding of PA to FnBPs.

The biocompatibility of wound dressings is essential. The toxicity of the materials was evaluated by using both direct contact culture and leachate culture of L929 cells. As shown in Figure S5A,B, Supporting Information, the FGMA/FG/PA hydrogel exhibit good biocompatibility. Then, the live‐dead staining of L929 cells further confirms that the hydrogel will not cause cell death (Figure S5C, Supporting Information).

### 2.4 Effect of Immune Regulation on Angiogenesis In Vitro

Due to the heterogeneous and plastic phenotype of macrophages, macrophages can usually polarize into the pro‐inflammatory phenotype M1, or the reparative and anti‐inflammatory phenotype M2.^[^ ^31^ ^]^ However, it is difficult for diabetic wounds to achieve the conversion of M1 to M2. The accumulation of M1 macrophages leads to ROS production and long‐term inflammation.^[^ ^32^ ^]^ Therefore, the potential of the hydrogel to scavenge ROS as a wound dressing was systematically tested. The ROS scavenging properties were verified by 1,1‐diphenyl‐2‐picrylhydrazine (DPPH radical), •OH (hydroxyl radical), and •O_2_^−^ (superoxide anion radical).^[^ ^33^ ^]^ The color of the solution gradually changed from dark to light with the increase of PA content, indicating that the FGMA/FG/PA hydrogel containing PA has significant radical scavenging ability (Figure S6A, Supporting Information). The scavenging rate of DPPH radicals is above 85% for PA content above 1% (Figure S6B, Supporting Information). Meanwhile, the FGMA/FG/PA hydrogels with PA content of 2% or more have excellent •OH and •O_2_^−^ scavenging effects, as shown in Figure S6C,D, Supporting Information. Intracellular ROS clearance was measured by the ROS indicator dichlorodihydro‐fluorescein diacetate (DCFH‐DA). Excess intracellular ROS was produced by stimulating cells with hydrogen peroxide (H_2_O_2_), causing DCFH‐DA oxidation to produce fluorescence.^[^ ^3^, ^34^ ^]^ The fluorescence positivity of Raw 264.7 cells treated with FGMA/FG hydrogel without PA is higher compared to the H_2_O_2_ stimulated group, while the fluorescence of Raw 264.7 cells treated with the FGMA/FG/PA hydrogel is significantly weaker, confirming the antioxidant ability of the FGMA/FG/PA hydrogels (Figure S7A,B, Supporting Information).

**Figure 4:**
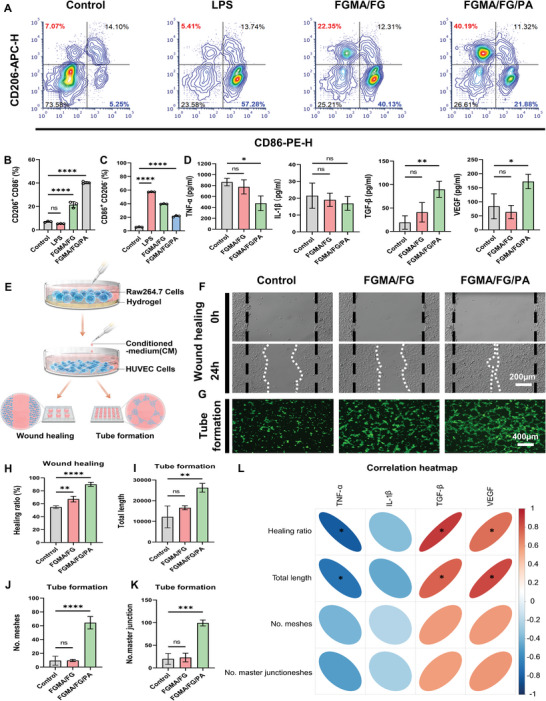
Effect of immune regulation on angiogenesis in vitro. A) Flow cytometry analysis of CD206 and CD86 expression of Raw 264.7 cells treated with PBS (control), LPS, FGMA/FG+LPS, FGMA/FG/PA+LPS. Statistical histogram of B) M1 (CD86+) or C) M2 (CD206+) phenotype macrophages ratio after being treated with PBS (control), LPS, FGMA/FG+LPS, FGMA/FG/PA+LPS. (****, *p* < 0.0001, *n* = 3). D) ELISA analysis of IL‐6, IL‐1*β*, TGF‐*β*, and VEGF (*, *p* < 0.05; **, *p* < 0.01, *n* = 3). E) Schematic diagram of exploring the effect of different hydrogels‐treated CMon migration and tube formation of HUVECs. F) Scratch assay images of HUVEC cultured in the medium supplementary with FGMA/FG and FGMA/FG/PA hydrogels. G) Representative images of tube formation after 3 h coculture. H) Healing rate of HUVEC cells in scratch assay (**, *p* < 0.01; ****, *p* < 0.0001, *n* = 3). I–K) In vitro tube formation results of HUVECs (**, *p* < 0.01; ***, *p* < 0.001; ****, *p* < 0.0001, *n* = 3). L) Correlation analysis of vascular regeneration indicators with inflammatory factors (*, *p* < 0.5).

Gelatin has been shown to exhibit anti‐inflammatory transforming ability,^[^ ^8b^ ^]^ and the anti‐inflammatory properties of PA have been reported more frequently.^[^ ^13b^ ^]^ It was hypothesized that the anti‐inflammatory effect of PA was achieved through immunomodulatory macrophage transformation. To verify this hypothesis, the immunomodulatory ability of the hydrogels was verified by co‐culturing Raw 264.7 cells with the FGMA/FG and FGMA/FG/PA hydrogels for 24 h, and the PBS‐treated group was used as a control group. The immunofluorescence staining of CD206, a characteristic marker of M2 macrophages, was performed to study the changes of cell phenotype after treatment with different hydrogels.^[^ ^35^ ^]^ Raw 264.7 cells co‐cultured with the FGMA/FG and FGMA/FG/PA hydrogels for 24 h show a stronger intensity of CD206 red fluorescence intensity (Figure S8, Supporting Information). Especially, the red fluorescence is significantly enhanced in the FGMA/FG/PA hydrogel group compared with the control group, indicating that the FGMA/FG/PA hydrogel activates the polarization of macrophages toward the M2 phenotype. The ability of the FGMA/FG/PA hydrogel to promote M1 macrophage polarization in vitro was then verified by stimulating macrophage polarization to the M1 phenotype using lipopolysaccharide (LPS) followed by treatment with FGMA/FG and FGMA/FG/PA hydrogels. The M1 and M2 phenotypes of macrophages were labeled with CD86 and CD206 respectively, and the conversion levels of macrophages in each group were detected by flow cytometry.^[^ ^36^ ^]^ The percentages of CD206^−^CD86^+^(M1) and CD206^+^CD86^−^(M2) macrophages in the control group are 5.56% and 6.97%, respectively, whereas the percentage of M1 macrophages is significantly increased to 57.34% after stimulation by LPS (**Figure** **4**A). After treatment of LPS‐stimulated polarized M1 macrophages with FGMA/FG/PA hydrogel, the percentage of M1 macrophages significantly decreased to 21.60% and the percentage of M2 macrophages increased to 40.35%. According to the statistical results in Figures 4B,C, the FGMA/FG/PA hydrogel can effectively promote the conversion of macrophage phenotype from M1 to M2 with better immunomodulatory ability.

Immunomodulation regulates vascular function through the secretion of various cytokines. After co‐culture with different hydrogels, the conditioned medium (CM) of macrophages^[^ ^37^ ^]^ was taken to test the secretion of relevant cytokines by Enzyme‐Linked Immunosorbnent Assay (ELISA). As shown in Figure 4D, the levels of pro‐inflammatory chemokine IL‐1*β* and tumor necrosis factor TNF‐*α* are lower in the FGMA/FG/PA hydrogel group compared with the control and FGMA/FG hydrogel groups. Meanwhile, transforming growth factor TGF‐*β* and vascular endothelial growth factor VEGF are more in the CM of macrophages co‐cultured with the FGMA/FG/PA hydrogel. This further suggests that the FGMA/FG/PA hydrogel is able to polarize macrophages to an M2 anti‐inflammatory phenotype, which is consistent with the results of flow cytometry, resulting in the release of factors that favor cell differentiation and angiogenesis‐related factors.

Human umbilical vein endothelial cells (HUVEC) were then cultured using the CM of different hydrogels‐treated macrophages, and cell migration and tube formation were assessed (Figure 4E). As shown in Figure 4F, the healing rate of HUVEC cells is significantly different after culturing with the CM in FGMA/FG/PA group compared to the control and FGMA/FG hydrogel groups (Figure 4H). In addition, the effect of immunomodulation on angiogenesis in vitro under different treatments was investigated by HUVEC cells tube formation assay.^[^ ^38^ ^]^ As shown in Figure 4G,I–K, the length of vessel formation, the number of mesh and the number of vessel formation are significantly greater in the FGMA/FG/PA group than in the control group. According to the correlation analysis (Figure 4L), the ability of CM of macrophages to promote the migration and tube‐formation of HUVEC cells is mainly related to the expression of TNF‐*α*, TGF‐*β*, and VEGF. In conclusion, these in vitro results suggest that the all‐natural FGMA/FG/PA hydrogel can alter the secretion of cytokines in the microenvironment through immunomodulation, significantly accelerating the migration and tube‐formation of HUVEC cells.

### 2.5 In Vivo Anti‐Infective and Immunomodulatory

**Figure 5:**
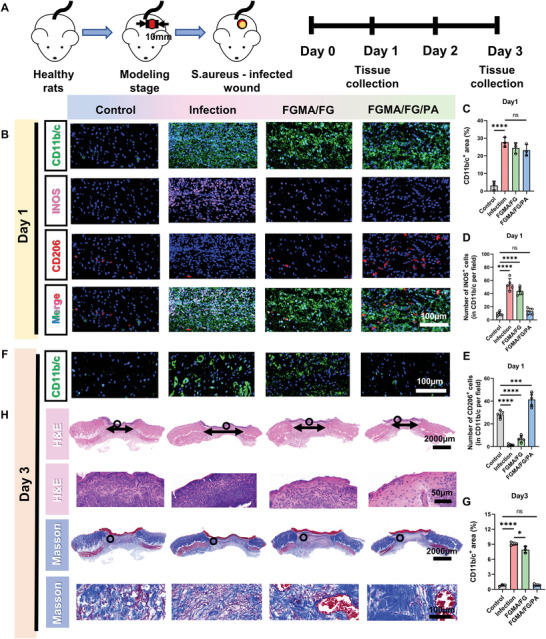
Effect of immune modulation on wound resistance to infection in Sprague Dawley rats. A) Schematic diagram of establishing an infectious wound and the timeline of animal experiments to test the therapeutic effect of hydrogels. B) Immunostaining of CD11b/c (green) shows the level of inflammation, CD11b/c (green) and iNOS (pink) immunostaining shows accumulation of M1 macrophages, CD11b/c (green) and CD206 (red) immunostaining shows accumulation of M2 macrophages at the wound bed on day 1. C) Statistical data of CD11b/c^+^ areas at the wound bed on day 1 (****, *p* < 0.0001, *n* = 3). Statistical data of D) iNOS and E) CD206 positive cells per in each group (***, *p* < 0.001; ****, *p* < 0.0001, *n* = 5). F) Immunofluorescence of CD11b/c (green) shows the level of inflammation at the wound bed on day 3. G) Statistical data of CD11b/c+ areas at the wound bed on day 1 (*, *p* < 0.05; ****, *p* < 0.0001, *n* = 3). H) H&E and Masson's trichrome staining on day 3.

The above results indicate that the FGMA/FG/PA hydrogel has good antibacterial properties, biocompatibility, ROS scavenging ability, and the ability to promote angiogenesis by altering the wound immune microenvironment. Therefore, the immunomodulatory and anti‐infective abilities of FGMA/FG/PA hydrogels were further investigated in vivo. As shown in **Figure** **5**A, a whole cortical *S. aureus*‐infected wound model was established.^[^ ^10b^ ^]^ Since the inflammatory phase of wound healing occurs during the first 3 days of infection, blood and tissue samples from days 1 and 3 were selected to study the inflammation and repair of the wound.

The immunofluorescence staining was performed for inflammatory cells CD11b/c, M1 macrophages (CD11b/c^+^iNOS^+^), and M2 macrophages (CD11b/c^+^CD206^+^).^[^ ^3c^ ^]^ The results verify the capacity of the FGMA/FG/PA hydrogel to modulate macrophage polarization in vivo, with the highest ratio of M2/M1 type macrophages among all groups (Figure 5B,D,E). However, there are no significant differences in CD11b/c^+^ inflammatory cells between the FGMA/FG/PA and the infected groups (Figure 5C). In acute infection, the matrix expresses a large amount of collagen to defend against infection. Immunofluorescence of type I collagen showed the strongest fluorescence intensity in the infected group and the weakest in the FGMA/FG/PA group (Figure S9A,B, Supporting Information). This demonstrates the ability of the FGMA/FG/PA group to resist infection. And at day 3, the CD11b/c+ inflammatory cells are significantly reduced in the FGMA/FG/PA group, as shown in Figure 5F,G. This illustrates that the FGMA/FG/PA hydrogel promotes immunomodulation of the infected wound and shortens the inflammatory phase (Figure S10, Supporting Information). The immunomodulatory ability of the FGMA/FG/PA hydrogel was further verified by ELISA testing of blood samples on the first day. The FGMA/FG/PA group shows higher expression of anti‐inflammatory factors IL‐10 and IL‐4, and lower expression of CPR (a non‐specific marker of acute inflammation) and pro‐inflammatory factors IL‐1*β*, IL‐6, and TNF‐*α* (Figure S11, Supporting Information), which are also consistent with the in vitro tests.

It is summarized that the FGMA/FG/PA hydrogel is able to modulate macrophage polarization in vivo and promote anti‐inflammatory processes, thus shortening the inflammatory phase. To further investigate its effect on the healing and regeneration process of infected wounds, histological analysis of the wounds on the third day was performed. The H&E staining images and statistical results of wound length show that the inflammatory cell infiltration is significantly reduced in the FGMA/FG/PA group and the wound length is similar to that of the control group (Figure 5H; Figure S12A, Supporting Information). This indicates that the infected wounds in the FGMA/FG/PA group had a shorter inflammatory phase due to the accelerated anti‐inflammatory process, which allows wound repair to enter the proliferative phase and accelerates wound contraction and healing. In addition, Masson trichrome staining also shows that the FGMA/FG/PA hydrogel can promote collagen formation and deposition (Figure 5H; Figure S12B, Supporting Information).

### 2.6 Diabetic Wound Immunomodulatory, Repair, and Remodeling In Vivo

**Figure 6:**
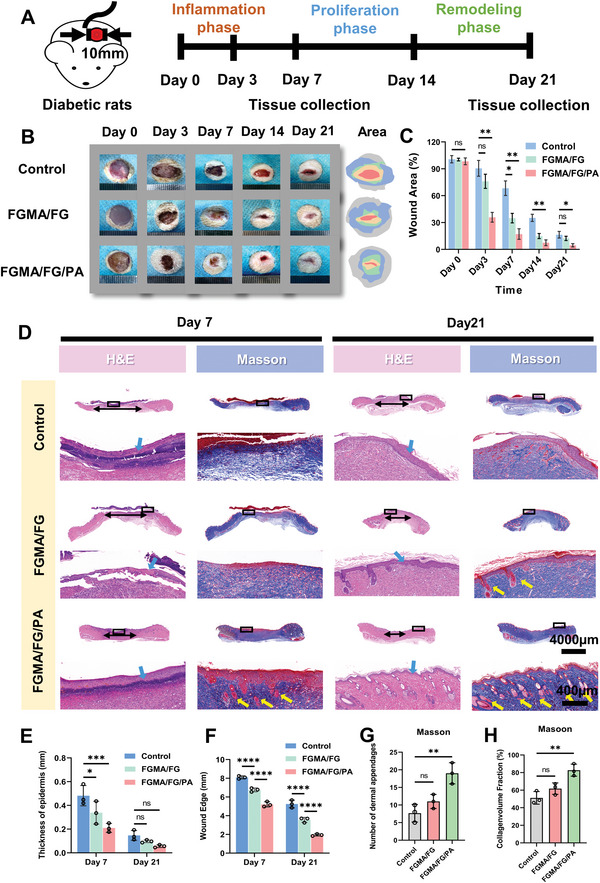
Effects of the all‐natural hydrogel in promoting diabetic wound healing in Sprague Dawley rats. A) Schematic diagram of establishing a diabetic wound and the timeline of animal experiments to test the therapeutic effect of hydrogels. B) Representative images of the wound at day 0, 3, 7, 14, 21 and the diagrams of time‐evolved wound areas. C) Statistical analysis of the wound area (*, *p* < 0.05; **, *p* < 0.01, *n* = 3). D) H&E staining and Masson's trichrome staining of wound tissues at various time points. Bule arrows indicate the re‐epithelialization area. Yellow arrows indicate newly formed dermal. E–F) Quantification of the epidermis thickness and length of wounds at day 7 and 21 (*, *p* < 0.05; ***, *p* < 0.001; ****, *p* < 0.0001, *n* = 3). G–H) Statistical analysis of collagen deposition and number of newly formed dermal at day 21 (**, *p* < 0.01, *n* = 3).

In vitro studies have shown that the all‐natural FGMA/FG/PA hydrogel has great promise for diabetic wound repair. Therefore, by administering streptozotocin (STZ) to Sprague Dawley rats and detecting changes in blood glucose daily, the diabetic rat model was successfully established when blood glucose levels were consistently >16.65 mmol L^−1^.^[^ ^12b^ ^]^ Then, whole wounds (10 mm in diameter) were created and treated with PBS, FGMA/FG and FGMA/FG/PA hydrogels, respectively. The effect of the FGMA/FG/PA hydrogel on accelerating diabetic wound healing was assessed by observing wound healing on distinct phases: inflammation, proliferation, and remodeling (**Figure** **6**A). As shown in Figure 6B, the wounds treated with the hydrogel healed faster than the control group. In particular, the healing rates of the FGMA/FG/PA hydrogel‐treated wounds on day 7 and day 21 are about 80% and 94%, respectively (Figure 6C), indicating that the FGMA/FG/PA hydrogel can effectively shorten the inflammatory phase to allow the wound to enter the proliferative phase of healing. The H&E staining results are consistent with the wound healing rate, and the FGMA/FG/PA hydrogel‐treated group shows better healing based on the epidermal thickness and wound length counted, approaching a state of complete healing at 21 days (Figure 6D–G).^[^ ^39^ ^]^ The proper collagen deposition and regeneration of dermal appendages are also important indicators for wound healing because of their ability to increase the tensile strength of the tissue and improve healing.^[^ ^12c^ ^]^ Therefore, the formation of new collagen was observed by Masson trichrome staining. The control group still has partial crusting at day 21. In contrast, the FGMA/FG/PA hydrogel‐treated wounds show a clear epidermal layer (Figure 6D) and have a higher fraction of dermal appendages and new collagen volume (Figure 6F,H). This indicates that the FGMA/FG/PA hydrogel‐treated wounds heal well at 21 days similar to healthy skin, and remodeling is completed.

**Figure 7:**
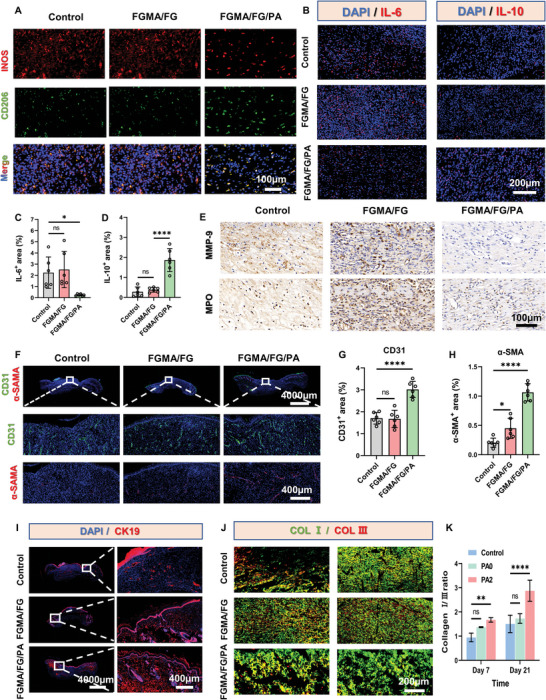
The effect of the all‐natural hydrogel on the three stages of wound healing. A) Immunofluorescence of iNOS (pink) immunostaining showed accumulation of M1 macrophages, and CD206 (red) immunostaining showed accumulation of M2 macrophages at the wound bed on day 7. B) Immunofluorescence staining results of pro‐inflammatory cytokines IL‐6 (red) and anti‐inflammatory cytokines IL‐10 (red) at day 7. C,D) Statistical data of IL‐6^+^ and IL‐10^+^ areas at the wound bed on day 7 (*, *p* < 0.05; ****, *p* < 0.0001, *n* = 5). E) Immunofluorescence staining results of CD31 (green) and *α*‐SMA (red) at day 21. F) Immunofluorescence staining results of CD31 (green) and *α*‐SMA (red) at day 21. G,H) Statistical data of CD31^+^ and *α*‐SMA ^+^ areas at the wound bed on day 21 (*, *p* < 0.05; ****, *p* < 0.0001, *n* = 5). I) Immunofluorescence of CK19 (red). J) Immunofluorescence of Collagen I and Collagen III at day 21. K) Collagen remodeling of every group at day 7 and 21 (**, *p* < 0.01; ****, *p* < 0.0001, *n* = 3).

In diabetic wounds, the inflammatory phase is prolonged due to the high glycemic environment in the blood, leading to excessive inflammation, accumulation of inflammatory cells, release of inflammatory factors, and difficulties in immune regulation.^[^ ^40^ ^]^ During the overlapping phases of inflammation and proliferation, we analyzed the effects of the all‐natural FGMA/FG/PA hydrogel on regulating macrophages heterogeneity, in addition to the effects on inflammatory cell accumulation and inflammatory factor secretion after immunomodulation. As shown in **Figure** **7**A, the percentage of iNOS (M1) in the FGMA/FG/PA hydrogel‐treated wound is significantly lower, while the percentage of CD206 (M2) is significantly higher, suggesting that the FGMA/FG/PA hydrogel can convert pro‐inflammatory M1 macrophages to anti‐inflammatory M2 macrophages. The expression of pro‐inflammatory factor IL‐6 and anti‐inflammatory factor IL‐10 also confirms these results (Figure 7B–D; Figure S13A, Supporting Information).^[^ ^41^ ^]^ To investigate the effect of the immunomodulation on the accumulation of inflammatory cells, the levels of matrix metalloproteinases MMP‐9 (activation and infiltration of inflammatory cells such as macrophages and neutrophils at the trabeculae leading to the secretion of MMPs) and myeloperoxidase MPO (a direct indicator of neutrophil infiltration) were determined at the trabeculae of each group (Figure 7E).^[^ ^40a^ ^]^ The expression of MPO and MMP‐9 at the trauma site is significantly lower in the FGMA/FG/PA hydrogel‐treated group compared to the control and FGMA/FG hydrogel‐treated groups, indicating that the FGMA/FG/PA hydrogel can reduce inflammation by regulating macrophages heterogeneity, increasing the conversion of M1 phenotype macrophages to M2 phenotype, and reducing the accumulation of inflammatory macrophages and neutrophils, etc.

During the proliferation phase, blood vessels have a crucial role in providing nutrients and oxygen to the cells, thus accelerating wound repair.^[^ ^12^, ^42^ ^]^ Therefore, this work used immunofluorescence to test angiogenesis at day 7.^[^ ^43^ ^]^ As shown in Figure S14A, Supporting Information, the green fluorescence of CD31 in neovascularization and red fluorescence of *α*‐SMA in neonatally matured vessels are more pronounced in the FGMA/FG/PA hydrogel‐treated wounds. Moreover, their *α*‐SMA and CD31 positive expression levels are the highest among all groups (Figure S14B,C, Supporting Information). As shown in Figure 7F–H, neovascularization is significantly increased in all groups at day 21, but the FGMA/FG/PA group has more mature vessels, longer vessels, and larger lumens. Vascular endothelial growth factor (VEGF) has also been widely used to evaluate vascular tissue regeneration.^[^ ^44^ ^]^ The highest VEGF expression levels are observed in the FGMA/FG/PA hydrogel‐treated group on days 7 (Figure S15A,B, Supporting Information), indicating that the FGMA/FG/PA hydrogel can promote vascular regeneration, which is also consistent with the results of in vitro tube formation experiments.

During the remodeling phase, collagen deposition, remodeling and follicle regeneration are the main indicators of the integrity of skin repair, with its ability to improve the tensile strength and epidermal integrity of the tissue.^[^ ^40^, ^45^ ^]^ Masson trichrome staining shows that the FGMA/FG/PA hydrogel‐treated wounds exhibit better collagen deposition and hair follicle regeneration at day 21 compared to the other two groups (Figure 6D). Therefore, the regeneration of wound hair follicles and collagen structures was further observed on day 21 using the hair follicle cell marker CK19 and type I and III collagen immunofluorescence staining. The results show that the FGMA/FG/PA hydrogel‐treated wounds have significantly stronger CK19 fluorescence and the highest percentage of type I/III collagen (Figure 7I,J). As shown in Figure 7K, the type I/III collagen ratio is low in the early stages of wound healing because the wound tissue is mainly immature type III collagen at this time. After tissue remodeling, type III collagen is slowly replaced by type I collagen because type I collagen has better tensile strength.^[^ ^46^ ^]^ This indicates that the FGMA/FG/PA hydrogel promotes collagen deposition and remodeling and increases follicle regeneration, bringing the restored tissue closer to healthy tissue.

## 3 Conclusion

In this study, an all‐natural hydrogel wound dressing with intrinsic immunomodulatory ability was developed for regulating macrophages heterogeneity targeting by increasing the conversion of pro‐inflammatory macrophages to anti‐inflammatory phenotypes. The all‐natural hydrogel exhibits good bioadhesive, ROS scavenging, and antibacterial properties, which can well protect the trauma surface from secondary damage. In addition, in vitro and in vivo experiments demonstrate that the prepared FGMA/FG/PA hydrogel has good anti‐infective and immunomodulatory abilities. Through immunofluorescence and flow testing of macrophages as well as pathological staining of tissues, biomarker detection, and immunofluorescence staining, the hydrogel is found to be able to regulate the immune microenvironment at the trauma site, shorten the inflammation period, promote vascular regeneration, and have good therapeutic effects on diabetic trauma. In summary, the developed all‐natural hydrogel can activate the immune regulation of macrophages with promising applications for diabetic wound repair and other immune‐related diseases treatment.

## Conflict of Interest

The authors declare no conflict of interest.

## Supporting information

Supporting Information

## Data Availability

The data that support the findings of this study are available from the corresponding author upon reasonable request.
