# Correction to “All‐Natural Immunomodulatory Bioadhesive Hydrogel Promotes Angiogenesis and Diabetic Wound Healing by Regulating Macrophage Heterogeneity”

**DOI:** 10.1002/advs.202404890

**Published:** 2024-07-01

*Advanced Science* **2023**, 10(13), https://doi.org/10.1002/advs.202206771

In the originally published paper, Figure 7A mistakenly included the wrong image due to a computer lag caused by extensive processing of immunofluorescence data from animal experiments. Consequently, the images of Control in Figure 7A were incorrect. We have replaced Figure 7A with the correct images. In addition, there were errors in the annotations to the pictures in Figures 7A, 7E, and 7J, which we have also corrected. The revised Figure 7 is shown below:

**Figure 7:**
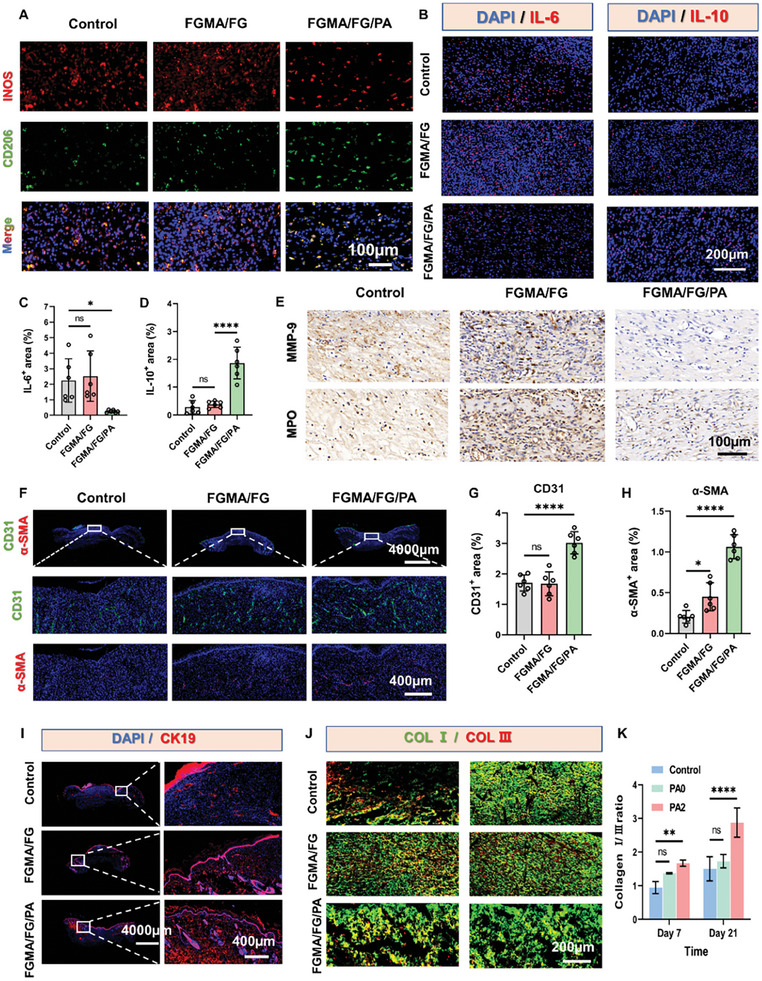
The effect of the all‐natural hydrogel on the three stages of wound healing. A) Immunofluorescence of iNOS (red) immunostaining showed accumulation of M1 macrophages, and CD206 (green) immunostaining showed accumulation of M2 macrophages at the wound bed on day 7. B) Immunofluorescence staining results of pro‐inflammatory cytokines IL‐6 (red) and anti‐inflammatory cytokines IL‐10 (red) at day 7. C,D) Statistical data of IL‐6^+^ and IL‐10^+^ areas at the wound bed on day 7 (^*^, *p* < 0.05; ^****^, *p* < 0.0001, n = 5). E) Representative immunohistochemical staining of MMP‐9 and MPO. F) Immunofluorescence staining results of CD31 (green) and α‐SMA (red) at day 21. G,H) Statistical data of CD31^+^ and α‐SMA ^+^ areas at the wound bed on day 21 (^*^, *p* < 0.05; ^****^, *p* < 0.0001, n = 5). I) Immunofluorescence of CK19 (red). J) Immunofluorescence of Collagen I and Collagen III at day 7 and day 21. K) Collagen remodeling of every group at day 7 and 21. (^**^, *p* < 0.01; ^****^, *p* < 0.0001, n = 3).

We apologize for the errors mentioned above. These corrections do not affect the results and conclusions presented in the published article.

