# Supplementary material for: All‐Natural Immunomodulatory Bioadhesive Hydrogel Promotes Angiogenesis and Diabetic Wound Healing by Regulating Macrophage Heterogeneity
Source: Adv Sci (Weinh). 2023 Mar 2;10(13):2206771. doi: 10.1002/advs.202206771 (PMC10161050; doi:10.1002/advs.202206771)
Supplement: Supplementary file 1 — Supporting Information [file ADVS-10-2206771-s001.pdf]

Supporting Information

All-natural immunomodulatory bioadhesive hydrogel promotes angiogenesis and diabetic wound healing by regulating macrophage heterogeneity

Ya-Jun Fu, Yi-Feng Shi, Li-Ya Wang, Yi-Fan Zhao, Rao-Kaijuan Wang, Kai Li, Shu-Ting Zhang, Xiang-Jun Zha, Wei Wang, Xing Zhao, and Wei Yang**

1. Experimental Section

Material: Gelatin from cold water fish skin (FG) was purchased from Sigma-Aldrich, methacrylic anhydride (MA, 94%) from Adamas, Phenyl-2,4,6-trimethylbenzoyl lithium phosphite（LAP）photoinitiator from Sigma-Aldrich, PBS buffer from Biosharp (PH = 7.0-7.2), protocatechuic aldehyde (PA, 99%) from Adamas. Propanetriol (GC, 99%) was purchased from Beijing Chemical Co.

Synthesis of FGMA: Methacrylated-modified gelatin was synthesized based on previous studies. Briefly, 10 g of gelatin (gelatin from cold water fish skin) was dissolved in 100 mL PBS solution (PH 7.0-7.2) and stirred with a magnetic stirrer at 300 rpm at 50 °C. Add 8 mL methacrylic anhydride (MA) to the gelatin solution dropwise with a micro syringe pump at 0.2 mL/min and react for 3 h. Then 100 mL PBS was added and stirred for 10 min to terminate the reaction. The reaction solution was poured into a dialysis bag (cut-off molecular weight 3500) and the water was changed every morning and evening for 7 days to remove the by-products and unreacted MA. Lastly, the dialysis solution was lyophilized for 2 days to generate white product via freeze drier and stored at -20 ℃. To determine the degree of methacrylation, the FG and FGMA were examined through ^{1}H-NMR and FTIR.

Characterization of FGMA

Fourier transform infrared (FTIR) characterization: The FGMA lyophilized hydrogel precursors were analyzed by Nicolet 6700 IR spectrometer in total reflection mode in the wave number range 4000-400 cm^{-1} with a resolution of 4 cm^{-1}.

Quantification of the degree of methacrylation of gelatin:

$$\text{percent methacrylation} = 1 - \frac{\text{A(lysinemethylene of FGMA)}}{\text{A(lysinemethylene of unmodified FGMA)}} \quad (1)$$

NMR hydrogen spectroscopy characterization: For ^{1}H-NMR, 6 mg of lyophilized FG and FGMA were dissolved in 0.6 mL of deuterium oxide (D_2O) at 37 °C and tested with 128 scans using a Bruker AV III HD 400 MHz nuclear magnetic resonance hydrogen spectrometer.

Synthesis of FG/PA: 10% wt of FG and 2% wt of PA were dissolved in DI Water at 40 °C to completely dissolve

FG and PA. Then dry the FG/PA.

Characterization of FG/PA

NMR hydrogen spectroscopy characterization: For ^{1}H-NMR, 6 mg of FG and FG/PA were dissolved in 0.6 mL of deuterium oxide (D_2O) at 37 °C and tested with 128 scans using a Bruker AV III HD 400 MHz nuclear magnetic resonance hydrogen spectrometer.

Fabrication of the FGMA/FG/PA Hydrogels: The formation of FGMA/FG/PA hydrogels relies on the generation of free radicals by photo-initiators under light to enable the double bond polymerization of FGMA. The prerequisite for photoinitiation is that the pre-gel solution has a good transparency. The addition of PA turned the solution to an opaque brown color, which prevented the light to penetrate the pre-gel solution. The reason for this phenomenon is presumed to be the reaction of PA with FG and FGMA. Since glycerol has three hydroxyl groups, it has stronger interactions with gelatin molecular chains than water. Therefore, glycerol was added in order to compete for the interaction between PA and gelatin. The transparency of the gels keeps increasing as the glycerol: water (G:W) ratio increases. At a G:W = 1:2, the transparency is fine and the photo-crosslinking formability is favorable. Therefore, 10% wt of FG and 0.5%, 1%, 2% and 3% wt of PA were dissolved in a co-solvent with a 1:2 mass ratio of glycerol and water at 40 °C to completely dissolve FG and PA. Secondly, 10 wt% of FGMA was added to the FG/PA solution to dissolve it completely. Finally, 0.5% wt of LAP (2,4,6-trimethylbenzoyl) was added to the solution and irradiated under 405 nm UV light for 5 s to make it light-cured. Prior to testing and use, the hydrogels were soaked in PBS solution to completely remove the glycerol, and the glycerol was considered completely removed if the PBS-soaked hydrogels could be freeze-dried. The PA content of FGMA/FG/PA hydrogel in the text is 2% if no special instructions are given.

Characterization of the FGMA/FG/PA Hydrogels

Morphology of the Hydrogels: The FGMA and FGMA/FG/PA hydrogels were quenched by immersion in liquid nitrogen for 3 min, and the quenched samples were lyophilized for 24 h to obtain SEM samples. The gel sections were gold sprayed and characterized using SEM (JSM-5900LV model, JEOL, Japan) with an electron accelerating voltage of 20 kV.

Rheological Properties of the Hydrogels: Preparation of 25 mm diameter hydrogel discs for rheological performance testing. The storage modulus (G′) and loss modulus (G″) were investigated by a dynamic rheometer, AR 2000ex, TA Instruments, USA. First perform a strain scan in the strain range of 0.01-1% to determine the linear elastic region, and then select the strain values within the linear elastic region to perform a frequency scan in the frequency range of 100-0.01 Hz.

Swelling property of the hydrogel: Cylindrical FGMA and FGMA/FG/PA hydrogel samples of 10 mm diameter and 5 mm height were prepared for swelling rate testing. First, the hydrogels were freeze-dried and the weight of the initial hydrogel (W_0) was recorded. The hydrogels were then immersed in PBS and the weight of the hydrogel, was recorded at 6h, 12h, 24h, 36h, 48h, 60h and 72h, denoted as W_i. Three parallel samples were used for testing. The swelling ratio was calculated using the following equation:

$$SR = \frac{W_i}{W_0} \tag{2}$$

In vitro degradability of hydrogel: The hydrogels FGMA and FGMA/FG/PA were prepared as cylindrical shapes of 5 mm height and 10 mm diameter. First, the initial weight of the freeze-dried hydrogel samples (Ms) was recorded; then the hydrogels were immersed in PBS containing type II collagenase (2 μg/mL). The weight of the dried sample (Md) was recorded at different time intervals. Three parallel samples were used for testing. The percentage mass of the remaining hydrogel was calculated using the formula:

$$\text{Mass remaining (\%)} = \frac{Md}{Ms} \times 100\% \tag{3}$$

Mechanical Characterization: To measure the interfacial toughness, bonded samples with a width of 1.5 cm were prepared, placed between two pieces of pigskin, pressed and then tested by a standard 180-degree peel test using a mechanical testing machine (500 N force measuring element). All tests were performed at a constant peel speed of 50 mm/min^{-1}. The measured force reaches a plateau when the peeling process enters a steady state. The interfacial toughness was determined by dividing the plateau force (for the 180-degree peel test) by the tissue sample width.

To measure the shear strength, bonded samples with a bond area of 1.5 cm wide and 1 cm long were prepared and subjected to standard tensile shear tests using a mechanical testing machine (500 N force measuring element). All tests were performed at a constant tensile speed of 50 mm/min^{-1}. The shear strength was determined by dividing the maximum force by the area of attachment.

Self-Healing of the Hydrogel: FGMA/FG/PA hydrogels were stained to obtain two different colors of hydrogels. The two hydrogels were then cut into two sections, placed together and waited for 2 min to ensure complete healing of the sections.

Antibacterial properties of Hydrogels

Bacterial activation: 100 uL of bacterial lyophilization solution (glycerol: bacterial solution = 1:1) was added to 10 mL of pound liquid medium, 200 rpm at 37 °C with shaking, and the bacteria were passaged every other day, and the method was the same as before, and the third generation was the working solution.

Agar diffusion method: Prepare 7 mm diameter FGMA/FG and FGMA/FG/PA hydrogel, sterilize and set aside; spread 200 uL 10^6 CFU/mL *S. aureus* (N3) in LB agar medium by pushing it evenly; place the sample on the plate and incubate it for 24 h at 37 °C in 5% CO_2 aerobic environment, take pictures and observe the diameter of the inhibition circle.

Plate colony counting: Co-culture 5×10^6 CFU/mL *Staphylococcus aureus* (N3) with the material for 8 h, 24 h, 48 h (1 mL of bacterial solution was added to each sample), after each time point, the co-culture solution was gently blown evenly, diluted 10^6 times and then 100 uL was taken in LB agar medium and coated evenly in LB agar medium with flat push, incubated at 37 °C in 5% CO_2 aerobic environment for 24 h in inverted position and then photographed for counting, each group 3 parallel replicates.

Co-culture 1.8×10^6 CFU/mL *E. coli* (N3) with the material for 8 h, 24 h and 48 h (1 mL of bacterial solution was added to each sample), after each time point, the co-culture solution was gently blown evenly, diluted 10^6 times and 100 uL was taken in LB agar medium and spread evenly, and then incubated for 24 h at 37 °C with 5% CO_2 in an aerobic environment and then photographed and counted, each group 3 parallel replicates.

$$\text{Bacteria inhibition rate} = \frac{\text{A}-\text{B}}{\text{A}} \times 100\% \quad (4)$$

A and B correspond to the number of colonies in the blank and material groups, respectively.

SEM observation of bacterial morphology:

5×10^6 CFU/mL of *S. aureus* or *E. coli* (N3) was co-cultured with the material for 8 h. The material was removed, and the floating bacteria were gently washed off with PBS; 4% paraformaldehyde fixed night, 50%, 75%, 90%, 95%, 100% anhydrous ethanol were dehydrated in turn, and the sample was sprayed with gold to make the sample.

5×10^6 CFU/mL *S. aureus* or *E. coli* (N3) was co-cultured with the material for 8 h, gently blow the co-culture solution, take 600 uL of the co-culture solution and add it onto the crawling film, dehydrate 4% paraformaldehyde-fixed night, 50%, 75%, 90%, 95%, 100% anhydrous ethanol in turn, spray the sample with gold, and observe the free bacterial morphology on the surface of the crawling film by electron microscopy.

PA release performance test: The FGMA/FG/PA hydrogels were immersed in PBS solution, and then collect the extracts at 8h, 24h, and 48h, changing the PBS solution at the same time. The extracts were tested by UV absorption spectrophotometer and the absorbance at 279 nm was obtained.

In vitro biocompatibility test: Mouse fibroblast cell line L929 was cultured at 37 °C in high glucose medium (DMEM, Gibco) supplemented with 10% fetal bovine serum (FBS, Gibco) and 1% Dual Resistancein (Gibco) a 5% CO_2 environment. The proliferation of L929 cells co-cultured in different hydrogels was examined in two ways

using CCK-8. Briefly, 3×10^4 cells were inoculated on hydrogels or 24-well plates, respectively, by direct contact co-culture on hydrogels and submerged culture, and incubated with CCK-8 reagent for 2 h after 24 h and 48 h. Optical density (OD) was measured at 450 nm, and all tests were repeated three times. L929 cells were then incubated directly on hydrogels for 24 h followed by live/dead staining to evaluate the cytotoxicity of the cells. Live cells were stained with calcein-AM (green fluorescence) and dead cells were stained with PI (red fluorescence).

Antioxidant efficiency of hydrogels

$\cdot O_2^-$ scavenging assay: Superoxide anion ($\cdot O_2^-$) scavenging activity was assessed using a superoxide anion assay kit (Nanjing Jiancheng Bioengineering Institute, Nanjing, China) according to the manufacturer's instructions. 1 mg of FGMA/FG/PA hydrogel containing different concentrations of PA was added to the working solution. After standing for 10 min, the absorbance at 550 nm was measured using an enzyme marker.

·OH scavenging assay: Hydroxyl radical (·OH) scavenging activity was assessed using a hydroxyl radical assay kit based on TMB colorimetric method (Nanjing Jiancheng Bioengineering Institute, Nanjing, China). After incubation at 37 °C for 1 min, Fenton reagent was added to each well and the absorbance at 550 nm was measured using an enzyme marker after standing for 20 min.

DPPH radical scavenging assay: The DPPH radical scavenging capacity assay kit (Nanjing Jiancheng Bioengineering Institute, Nanjing, China) was used to determine the radical scavenging capacity based on the reduction of DPPH radicals by PA. DPPH working solution was prepared according to the manufacturer's instructions. 1 mg of FGMA/FG/PA hydrogel containing different concentrations of PA was added to the diluted DPPH working solution. After incubation at room temperature for 30 min, the scavenging efficiency of the hydrogels for DPPH was determined by measuring the absorbance at 517 nm.

$$\text{scavenging rate} = \frac{A-B}{A} \times 100\% \tag{5}$$

where A, B are the absorption of the blank and the absorption of the hydrogel respectively.

Intracellular ROS scavenging ability: 8×10^4 cells/well macrophages were inoculated in 24-well plates with high sugar medium containing DMEM 10% FBS and 1% double antibodies, incubated at 37°C in 5% CO_2 for 24 h. The medium was aspirated and washed 3 times using PBS solution gently, and then material extracts soaked for 24 h using DMEM medium were added and treated with H_2O_2 (100 μM) for 12 h. The solution was then aspirated, washed 3 times using PBS, and DCFH-DA (5 μM) (Reactive Oxygen Species Assay Kit, Beyotime Biotechnology) was added for 30 min before detecting ROS production in macrophages by Leica DMi8 inverted fluorescence

microscope.

In Vitro Macrophage Phenotype Regulation of the Hydrogels: Raw 264.7 was seeded at 2× 10^5 cells/well in 6-well plates, hydrogel protoplasts of 10 mm diameter were added, and immunofluorescence staining for CD206, a marker of M2 macrophages, was performed 24 h later. Briefly, macrophages were fixed with fixative for 20 min at 4 °C, broken with 1× membrane breaking solution, and then the cells were incubated with CD206 antibody overnight at 4 °C, and DAPI was used to visualize the nuclei. Images were obtained using a Leica inverted immunofluorescence microscope.

Flow cytometry testing was performed as follows: First, Raw 264.7 cells were inoculated in 6-well plates and incubated at 37°C with 5% CO_2 for 24 h. After that, serum-free DMEM containing 1ug/mL lipopolysaccharide (LPS) was added to the LPS stimulation group and incubated for 12 h to polarize macrophages to the M1 phenotype. After 12 h, the LPS solution was removed and washed with PBS. DMEM was added to the LPS group, and FGMA/FG and FGMA/FG/PA hydrogel were added to the material group, respectively. The Raw 264.7 cells in the control group was always cultured with DMEM medium. After 12h incubation, the supernatant was removed and the cells were collected after 2 gentle rinses using PBS. Live dead staining antibody and CD86 antibody were added first and incubated for 30 min at 4°C. Then the supernatant was removed by centrifugation, and excess antibody was washed off by adding PBS. The fixative was added and incubated for 20 min and then the membrane breaking solution was added for membrane breaking. After membrane breaking, CD206 antibody was added and incubated at 4°C for 30min, and then detected by flow cytometry. The above eBioscience™ Intracellular Fixation & Permeabilization Buffer Set, CD86 (B7-2) Monoclonal Antibody (GL1), PE, eBioscience™ and CD206 (MMR) Monoclonal Antibody (MR6F3), APC, eBioscience™ were purchased from Thermo Fisher Scientific.

Detection of inflammatory cytokines in conditioned media: 8×10^4 Raw264.7 cells were inoculated in 24-well plates containing different hydrogels of 10 mm diameter and then co-cultured for 24 h. The supernatant was then collected by centrifugation at 1000 rpm speed for 5 min, called conditioned medium. The conditioned medium was then tested by the Inflammatory Cytokine Assay ELISA kit (Thermo Fisher Scientific, USA).

HUVEC Cell Migration and Tube Formation Assessment

Cell migration assessment: 6-well plates were inoculated with 6×10^5 HUVEC cells per well, and after the cells grew to full size, they were scratched with a 200 μL pipette tip using a straightedge aid, and after scratching, the plates were washed three times with PBS and incubated with conditioned medium (CM) obtained by co-culture of different hydrogels with macrophages for 24 h. 0 and 24 h were photographed under a Leica DMi8 inverted fluorescence microscope.

Cell tube formation assessment: Firstly, matrix gel (Shanghai Nova Pharmaceutical Technology Co.) and DMEM medium were added to 48-well plates at a volume fraction of 1:1 and incubated at 37 °C for 45 min~1 h. Then 1×10^4 HUVEC cells were inoculated on the surface and 300 uL of different CM was added to each well. 3 h of incubation at 37 °C was used to take pictures using a Leica DMi8 inverted fluorescence microscope.

In vivo healing effect of hydrogel on infected wounds: All animal experiments were approved by the Institutional Review Board of West China Hospital, Sichuan University. Rats were habituated for 1 week prior to surgery. The rats were anesthetized by intraperitoneal injection of pentobarbital, and then the dorsal region of the rats was shaved and disinfected with iodophor before further surgery. Two full-length wounds of approximately 10 mm in diameter were made on each side of the midline of the rats. Subsequently, 10 uL of *S. aureus* (1×10^8 CFU/mL) was injected into the wound to establish infection. One group was treated by PBS, one group was treated by FGMA/FG hydrogel, and the third group was treated by FGMA/FG/PA hydrogel. Each group contains 5 rats. The rats were executed on the first and third days, and the blood and tissues were collected for ELISA, histological examination and immunofluorescence staining.

ELISA: One-day infected rats were anesthetized with pentobarbital, their chest skin was cut open, blood was taken from their hearts, and their blood was centrifuged at 3500 rpm before the supernatant was taken for detection using an ELISA test kit for inflammatory factors (Thermo Fisher Scientific, USA).

Histological examination and immunofluorescence staining: Wound tissues were collected at day1 and day3 after the model induction and fixed with paraformaldehyde (4% in PBS), embedded in paraffin wax and cut into 5 μm slices for following hematoxylin-eosin (H&E) staining, and immunofluorescence staining. H&E, and immunofluorescence staining were performed according to standard manufacturer's instruction protocols. Sections were deparaffinized with xylene and rehydrated in graded ethanol. Sections were microwaved in 10 mM sodium citrate and then incubated with primary antibodies overnight at 4 °C and the secondary antibodies for 30 min at 37 °C.

Wound tissues were fixed in 4% formaldehyde for paraffin embedding. Tissue sections (5 μm) were mounted on slides for histological analysis. Hematoxylin and eosin staining (H&E) was performed to measure wound closure areas. masson's trichrome staining was used to quantify collagen deposition. Immunofluorescence staining for type I collagen and CD11b/c, iNOS and CD206 was performed at days 1 and 3 of model establishment to observe macrophage infiltration and differentiation at the wound site.

In vivo healing effect of hydrogel on diabetic wounds: Prepared streptozotocin (STZ) solution 60 mg/kg was injected intraperitoneally into male SD rats weighing 200-250 g to induce type 1 diabetes mellitus. Rats with blood

glucose levels ≥16.7 mmol/L were considered successfully established 1 week after injection and were randomly divided into 3 groups. Two full skin wounds on the upper back were caused with a 10 mm caliber circular punch. The wounds were covered with PBS (control group), FGMA/FG, and FGMA/FG/PA hydrogel. Photographs of the trauma surfaces were taken after 3, 7, 14, and 21 days of treatment, and the trauma areas were calculated using Image J (n=3 per group). Meanwhile, at day 7 and 21, the rats were executed. Traumatic tissues were collected for histological examination and immunofluorescence staining.

The wound closure area was calculated by Image J. Wound contraction (%) was calculated using the equation: Wound contraction = (area (0 day)-area (n day))/ (area (0 day)) ×100%.

Wound tissues were fixed in 4% formaldehyde for paraffin embedding. Tissue sections (5 μm) were mounted on slides for histological analysis. Hematoxylin and eosin staining (H&E) was performed to measure wound closure areas. masson's trichrome staining was used to quantify collagen deposition. Immunohistochemical staining for MPO and VEGF, and immunofluorescence staining for IL-6, IL-10, iNOS, and CD206 were performed at day 7 of model establishment to observe macrophage infiltration and differentiation at the wound site. Wound healing was then assessed by detecting immunofluorescence for CD31, α-SMA, VEGF, CK19, and type I and type III collagen on days 7 and 21, respectively. The staining was quantified using Image J.

Statistical Analysis: All data were presented as means ± SD. Statistical analysis was performed between groups using one-way analysis of variance (ANOVA). A value of $P < 0.05$ was considered statistically significant.

2. Supporting Results

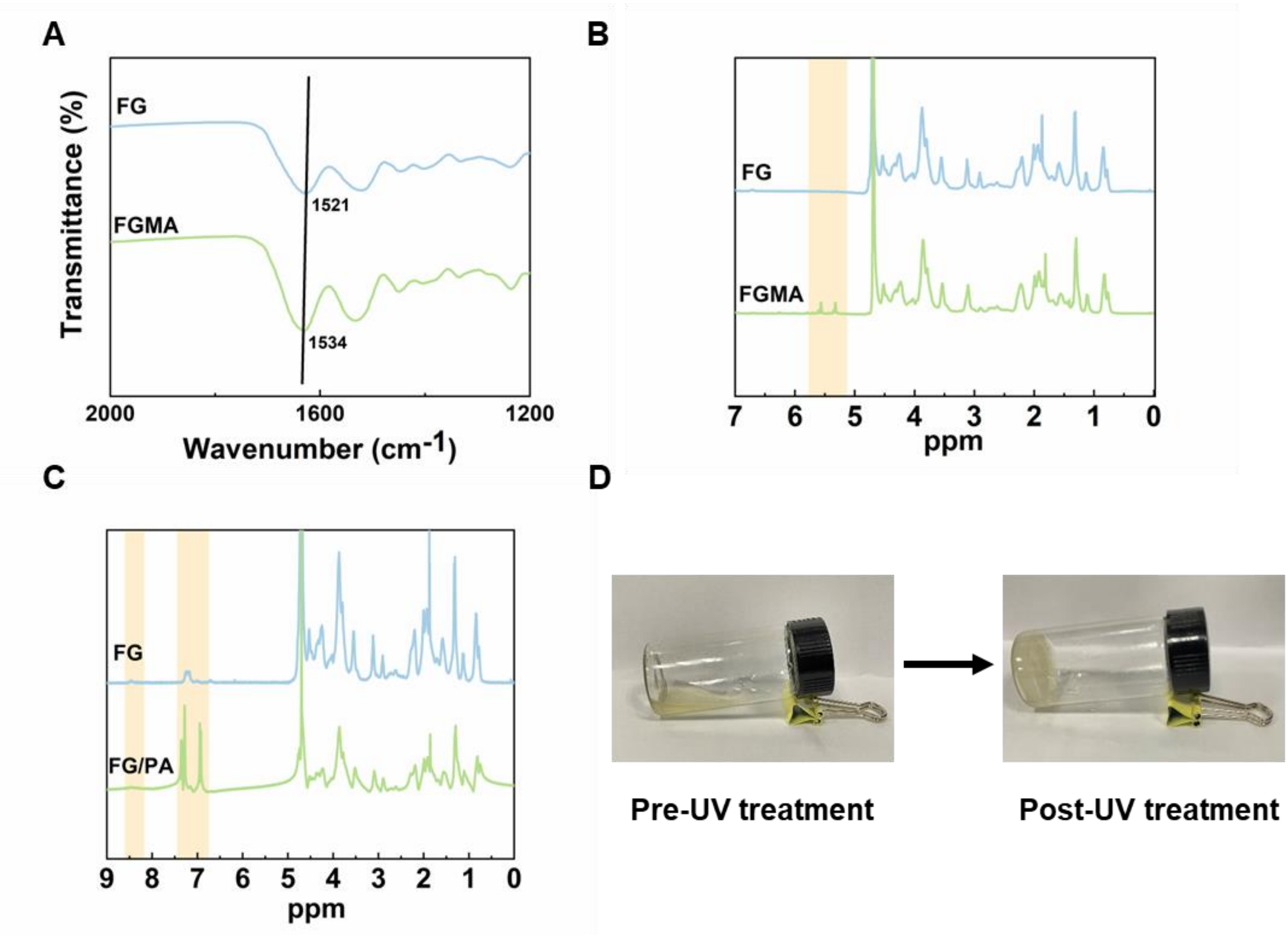

Figure S1. Preparation of the all-natural FGMA/FG/PA hydrogel. (A) FTIR spectra of FG, FGMA, the characteristic absorption peak at 1521 cm^{-1} in FG had occurred a blue shift, which indicated the successful modification of MA with FG. (B) ^{1}H-NMR spectra of FG and FGMA. Peaks corresponds to acrylic protons of methacrylamide groups. (C) ^{1}H-NMR spectra of FG and FGPA, indicating that the schiff base reaction can take place between PA and FG. (D) Photos of FGMA/FG/PA solution and hydrogel after UV crosslinked for 5 s.

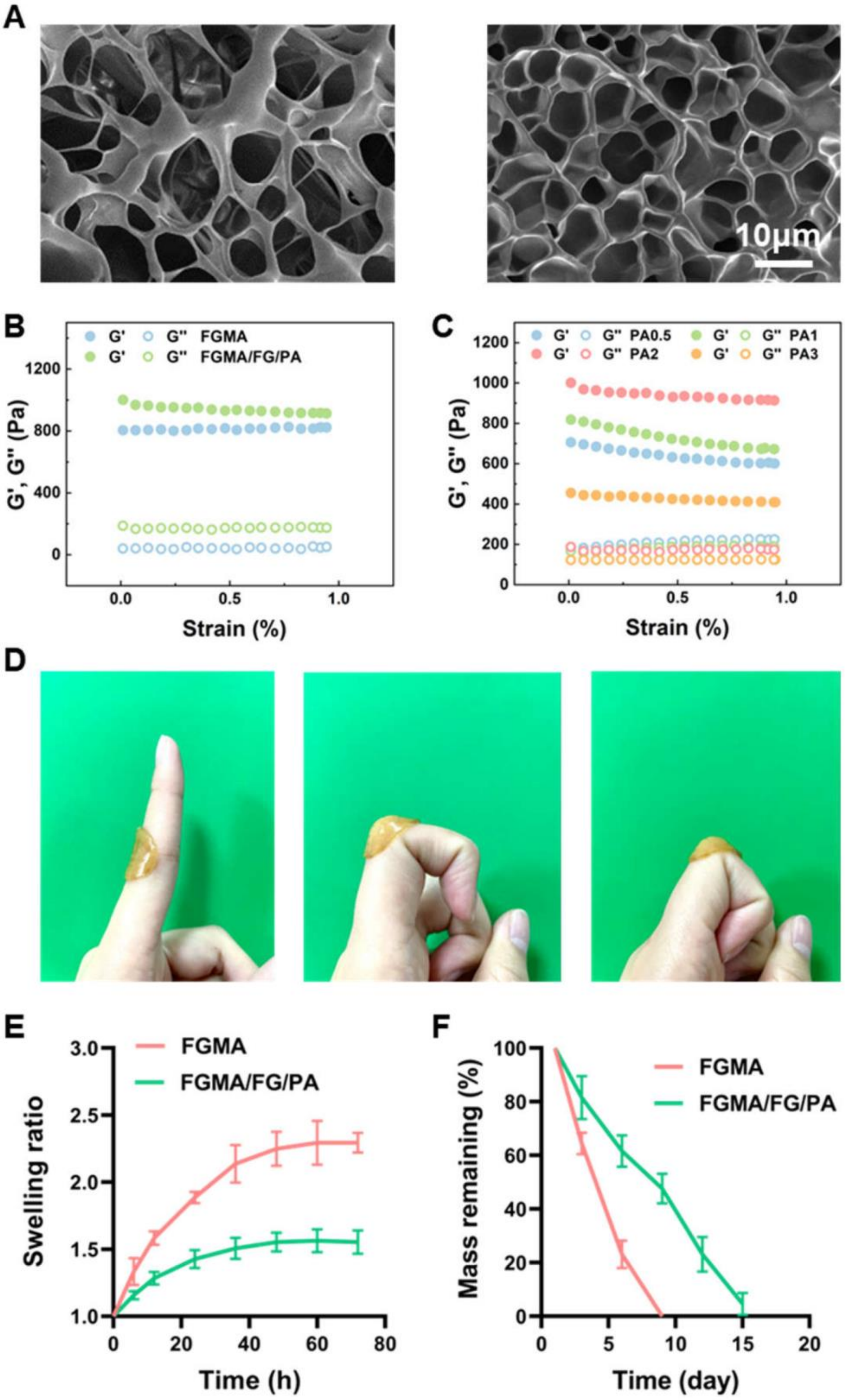

Figure S2. Characterization of the all-natural hydrogel. (A) SEM of the cross section of the FGMA, FGMA/FG/PA hydrogels (scale bar: 10 μm). (B) Strain-scan curve in the rheological characterization of FGMA, FGMA/FG/PA hydrogels. (C) Strain-scan curve in the rheological characterization of FGMA/FG/PA hydrogels at different PA concentrations. (D) Photographs of FGMA/FG/PA hydrogel changes with finger bending. (E) Swelling ratio of the hydrogels in phosphate buffered solution (PBS, pH = 7.4). (F) Degradation behavior of the hydrogels (FGMA and FGMA/FG/PA) in the presence of 2 μg/mL type II collagenase.

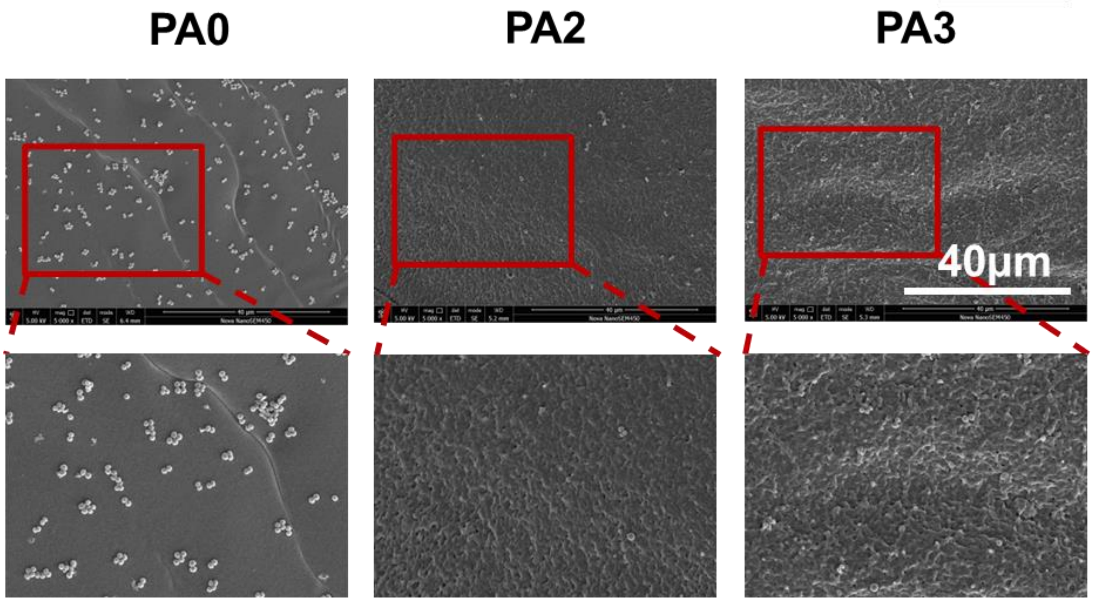

Figure S3. *S. aureus* adhesion on the surface of different hydrogel films (0%, 2%, 3% PA complex).

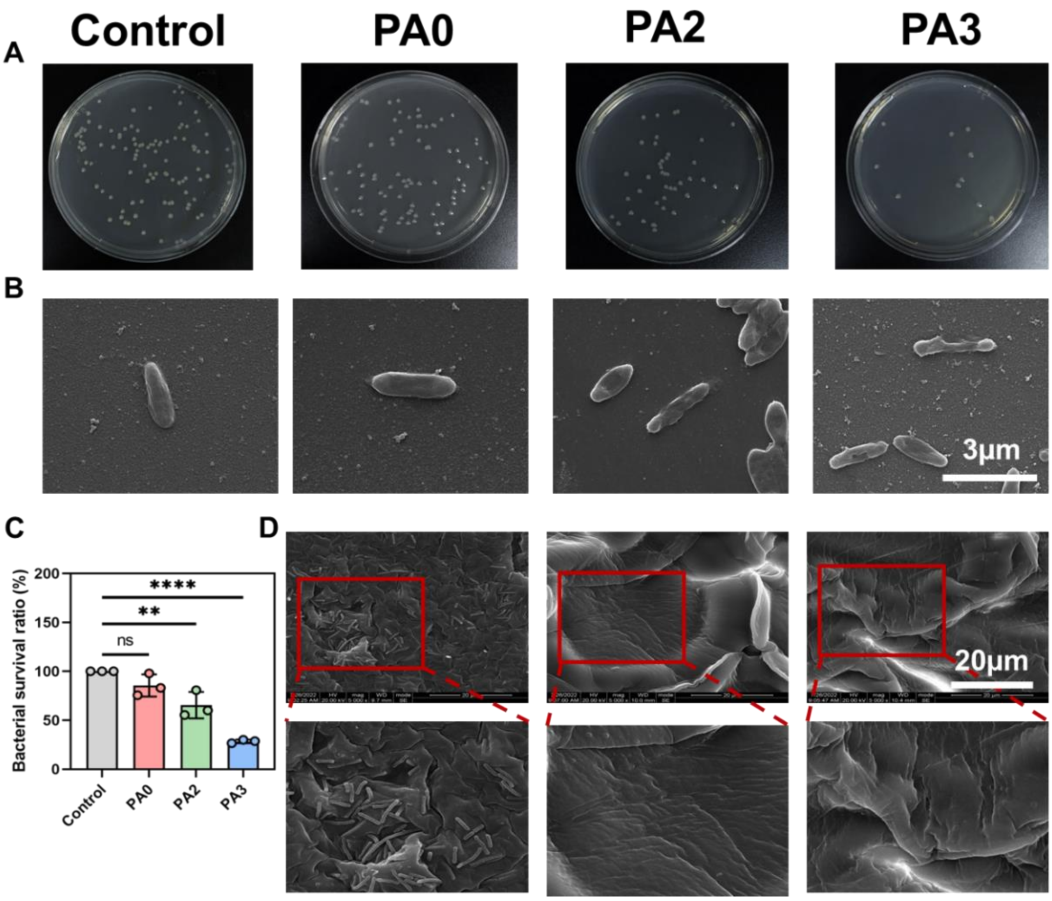

Figure S4. Antibacterial performance of hydrogel against *E. coli*. (A) Image of bacteria clones on culture plates against *E. coli* after incubation for 8 h at 37 °C and (B) SEM images of bacteria morphology (scale bar: 3 μm) with PBS and FGMA-FG-PA hydrogel (0%, 2%, 3% PA complex), respectively (n=3). (C) Bacterial viability against *E. coli* after incubation for 8 h at 37 °C (**, $p < 0.01$; ****, $p < 0.0001$, n=3). (D) Bacterial adhesion on the surface of different hydrogel films (0%, 2%, 3% PA complex), respectively (scale bar: 20 μm).

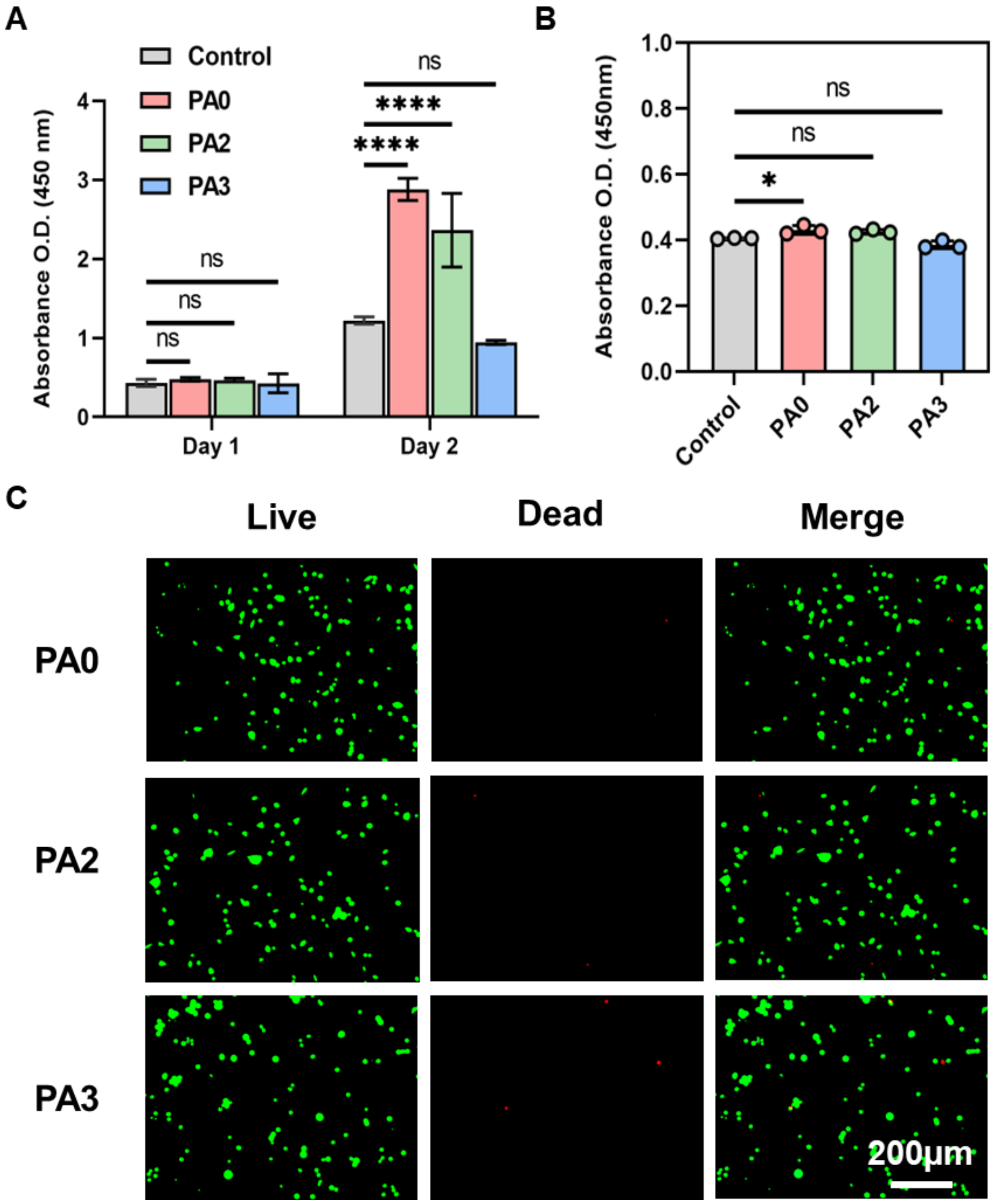

Figure S5. Biocompatibility of the all-natural hydrogels. (A) Cell viability of L929 cells treated with hydrogel extracts for 24 h and 48 h (****, $p < 0.0001$, n=3). (B) Cytotoxicity of hydrogel for L929 cells after co-incubation with 24 h using CCK-8 method (*, $p < 0.05$, n=3). (C) Live/dead staining of L929 cells after co-incubation with hydrogel for 24 h (scale: 200 μm).

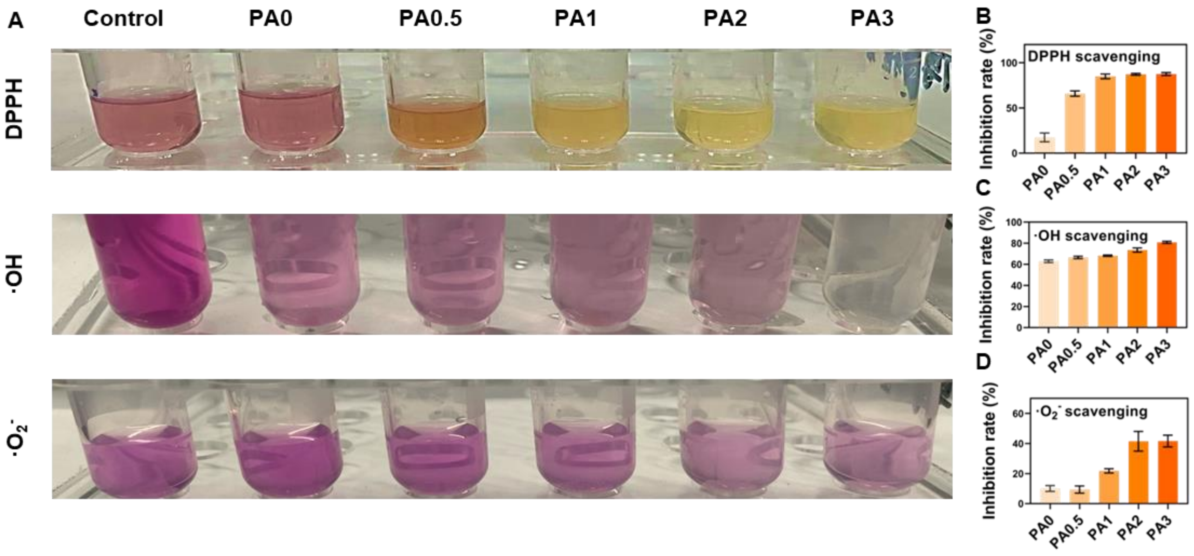

Figure S6. ROS-scavenging property of the all-natural hydrogel *in vitro*. (A) Image of DPPH, •OH and $•O_2^-$ scavenging results. (B-D) ROS inhibiting efficiency of FGMA/FG/PA at different PA concentrations for DPPH free radical, •OH and $•O_2^-$, respectively.

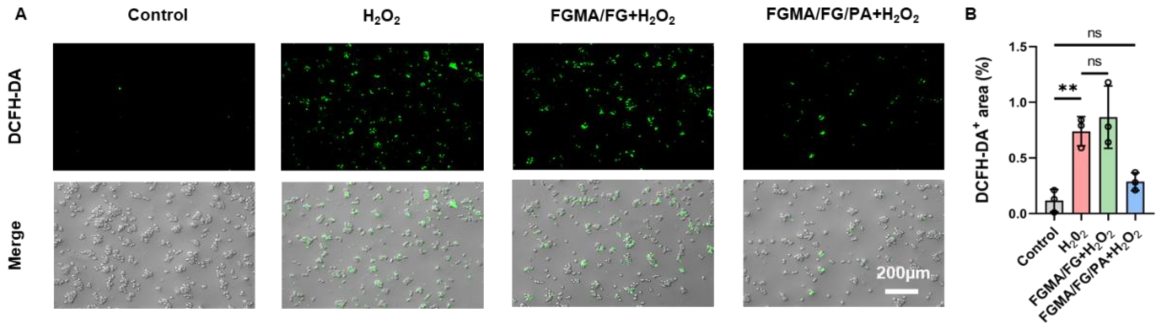

Figure S7. Intracellular ROS scavenging capacity. (A) Intracellular ROS-scavenging performance of FGMA/FG and the FGMA/FG/PA hydrogel (scale: 200 μm). (B) Statistical data of DCFH-DA$^+$ areas (**, $p < 0.01$, n = 3).

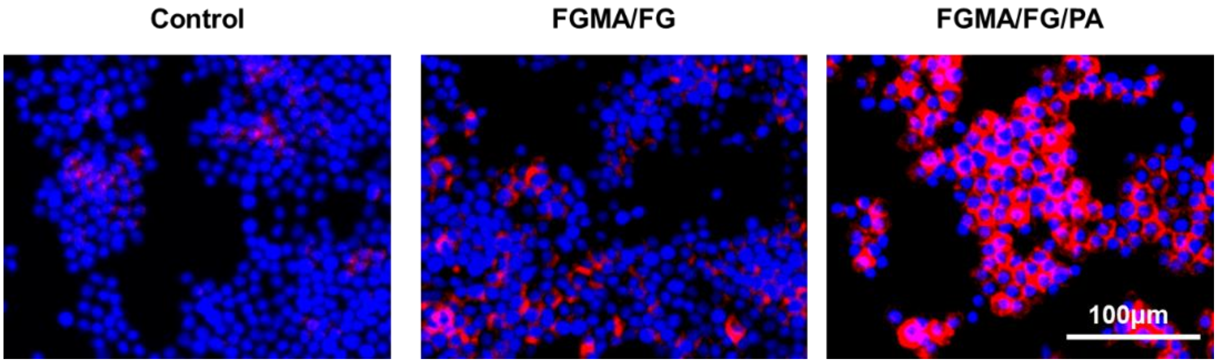

Figure S8. Representative images of Raw 264.7 cells treated by PBS, FGMA/FG, FGMA/FG/PA hydrogel for 24 hours (red, CD206; blue, cell nuclear).

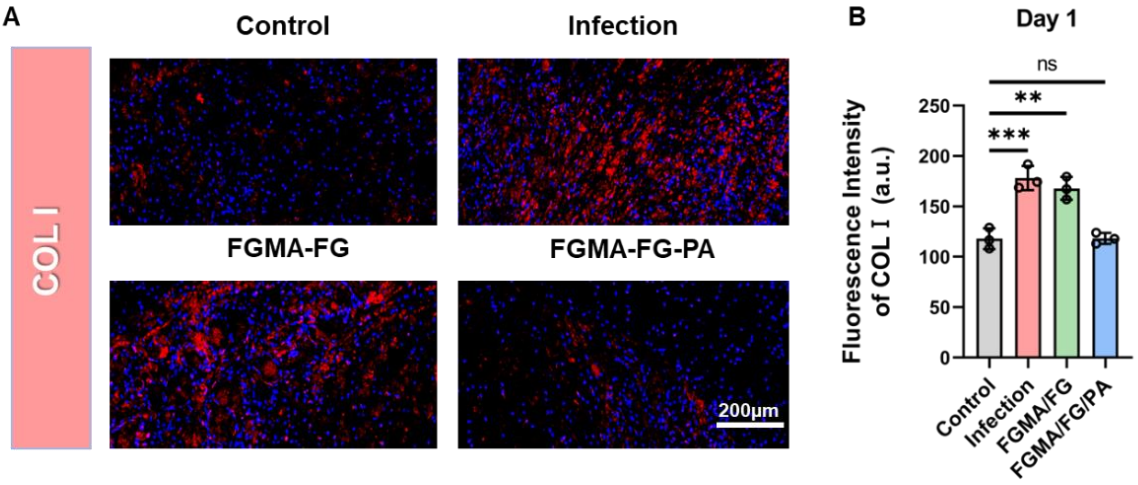

Figure S9. (A) Immunofluorescence of COL I on day 1. (B) Histogram of the fluorescence intensity of COL I in the different groups (**, $p < 0.01$; ***, $p < 0.001$, n=3).

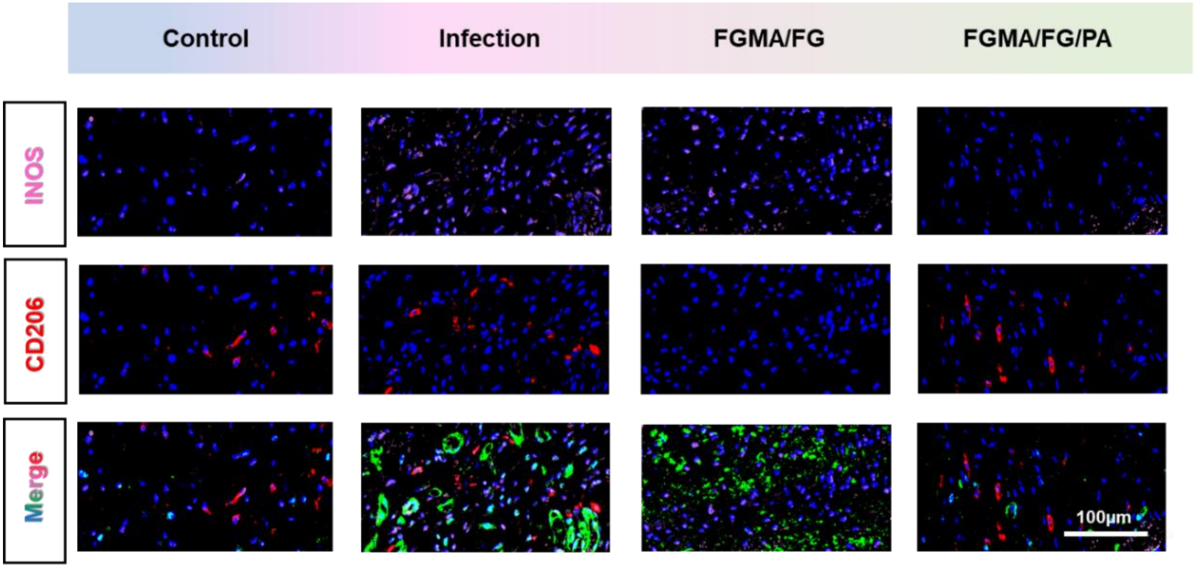

Figure S10. Immunofluorescence of CD11b/c (green) and INOS (pink) immunostaining showed accumulation of M1 macrophages, CD11b/c (green) and CD206 (red) immunostaining showed accumulation of M2 macrophages at the wound bed on day 3.

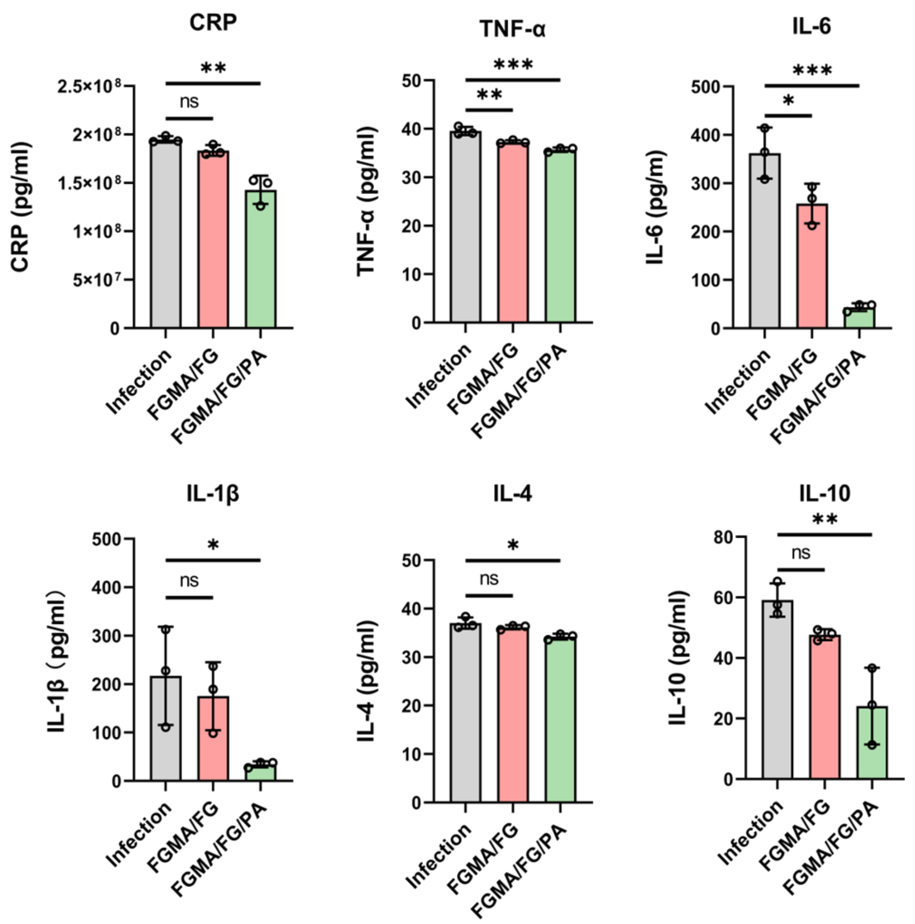

Figure S11. ELISA analysis of CRP, TNF-α, IL-6, IL-1β, IL-4, and IL-10 (*, $p < 0.05$; **, $p < 0.01$; ***, $p < 0.001$, n=3).

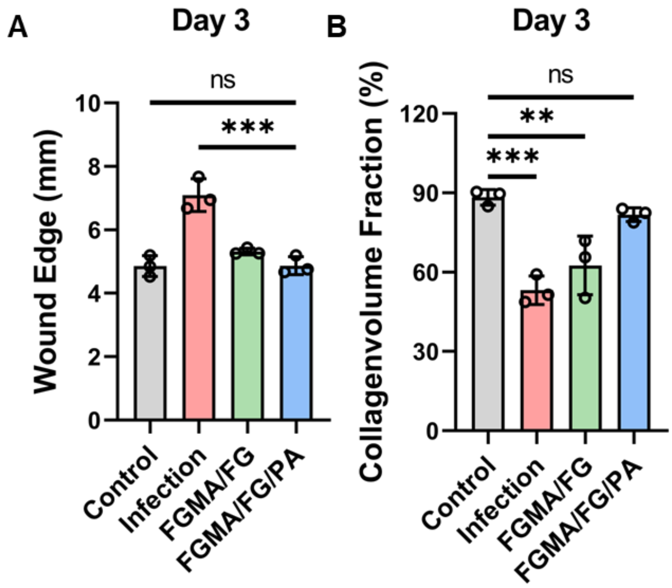

Figure S12. Quantification of the (A) wound edge and (B) collagenvolume fraction on day 3.

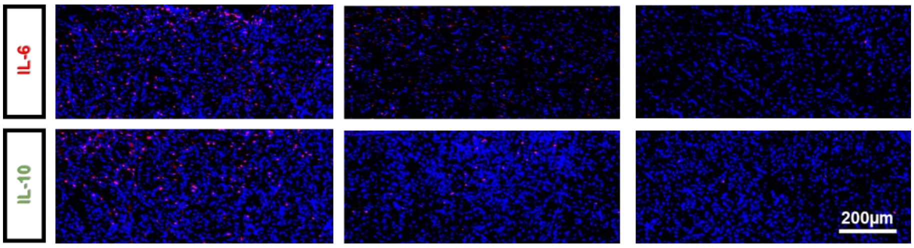

Figure S13. Immunofluorescence staining results of pro-inflammatory cytokines IL-6 (red) and anti-inflammatory cytokines IL-10 (red) at day 21.

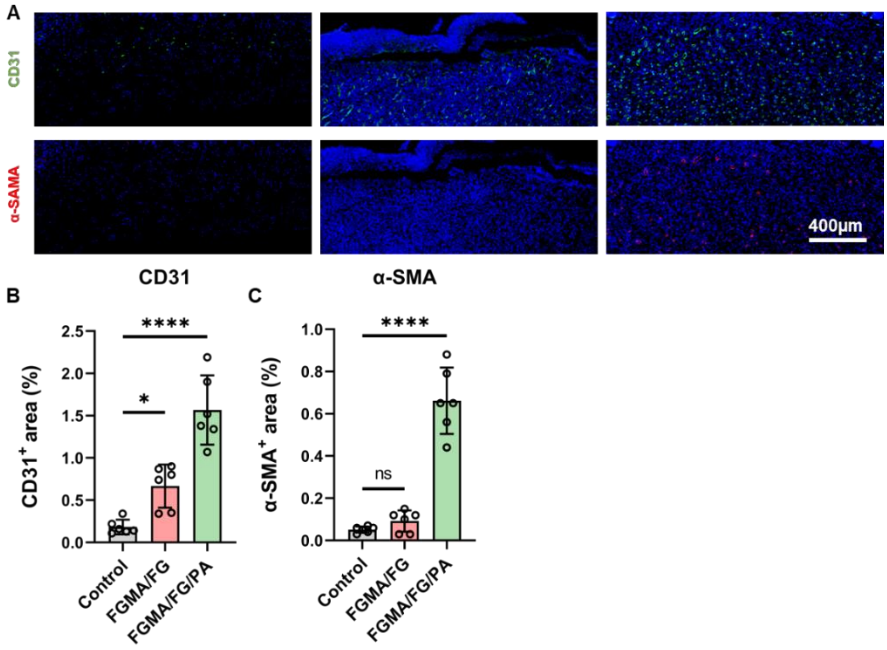

Figure S14. (A) Immunofluorescence staining results of CD31 (green) and α-SMA (red) at day 7. (B-C) Statistical data of CD31^{+} and α-SMA^{+} areas at the wound bed on day 7 (*, $p < 0.05$; ****, $p < 0.0001$, n=5).

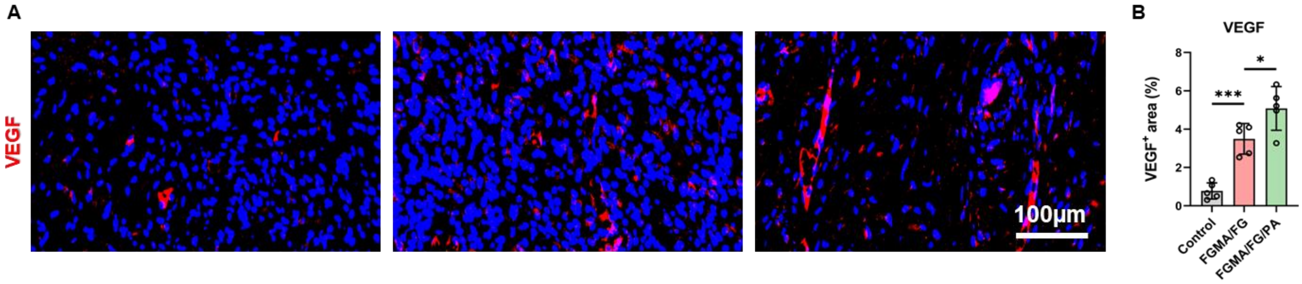

Figure S15. (A) Immunofluorescence staining results of VEGF at day 7. (B) Statistical data of VEGF^{+} area at the wound bed on day 7 (*, $p < 0.05$; ***, $p < 0.001$, n=5).
